# Clinical document corpora—real ones, translated and synthetic substitutes, and assorted domain proxies: a survey of diversity in corpus design, with focus on German text data

**DOI:** 10.1093/jamiaopen/ooaf024

**Published:** 2025-05-14

**Authors:** Udo Hahn

**Affiliations:** Institute for Medical Informatics, Statistics and Epidemiology (IMISE), University of Leipzig, D-04107 Leipzig, Saxony, Germany

**Keywords:** natural language processing, clinical text corpora, medical text corpora, German language

## Abstract

**Objective:**

We survey clinical document corpora, with a focus on German textual data. Due to rigid data privacy legislation in Germany, these resources, with only few exceptions, are stored in protected clinical data spaces and locked against clinic-external researchers. This situation stands in stark contrast with established workflows in the field of natural language processing, where easy accessibility and reuse of (textual) data collections are common practice. Hence, alternative corpus designs have been examined to escape from data poverty. Besides machine translation of English clinical datasets and the generation of synthetic corpora with fictitious clinical contents, several types of domain proxies have come up as substitutes for real clinical documents. Common instances of close proxies are medical journal publications, therapy guidelines, drug labels, etc., more distant proxies include medical contents from social media channels or online encyclopedic medical articles.

**Methods:**

We follow the PRISM (Preferred Reporting Items for Systematic reviews and Meta-analyses) guidelines for surveying the field of German-language clinical/medical corpora. Four bibliographic databases were searched: PubMed, ACL Anthology, Google Scholar, and the author’s personal literature database.

**Results:**

After PRISM-conformant identification of 362 hits from the 4 bibliographic systems, the screening process yielded 78 relevant documents for inclusion in this review. They contained overall 92 different published versions of corpora, from which 71 were truly unique in terms of their underlying document sets. Out of these, the majority were clinical corpora—46 real ones from which 32 were unique, 5 translated ones (3 unique), and 6 synthetic ones (3 unique). As to domain proxies, we identified 18 close ones (16 unique) and 17 distant ones (all of them unique).

**Discussion:**

There is a clear divide between the large number of non-accessible real clinical German-language corpora and their publicly accessible substitutes: translated or synthetic datasets, close or more distant proxies. So, at first sight, the data bottleneck seems broken. Intuitively, yet, differences in genre-specific writing style, lexical or terminological diction, and required medical background expertise in this typological space are also obvious. This raises the question how valid alternative corpus designs really are. A systematic, empirically grounded yardstick for comparing real clinical corpora with those suggested substitutes and proxies is missing until now.

**Conclusion:**

The extreme sparsity of real clinical corpora in almost all non-Anglo-American countries worldwide, Germany in particular, has triggered an active search for alternative, publicly accessible data resources laid out in this survey. However, the utility of these substitutes compared with real clinical corpora and their semantic and genre-specific distance to real clinical corpora is still under-researched so that their value remains to be assessed properly. Furthermore, corpus descriptions are often incomplete with respect to relevant descriptional attributes. This paper bundles these observations and proposes a template for a so-called corpus card to improve adequate corpus documentation.

## Background

Corpora are collections of so-called *unstructured* textual, audio, or visual data in contrast to structured, mostly tabular, information stored in databases or spreadsheets. Whereas structured data are readily interpretable and thus actionable by computers, unstructured data are not. To computationally interpret unstructured data, language models are automatically learned, which capture and represent the data’s structure and contents so that computers can reason on the models’ representation structures. This learning process is either organized in an unsupervised way, just relying on typically huge masses of raw corpus data and the distributional patterns they embody, or in a (semi-)supervised manner where metadata attached to the raw corpus data explicitly inform the machine learning engine with crucial (syntactic and) semantic interpretation hints.

Such metadata are usually supplied by humans as the result of annotation processes that lead to *gold standard* data (so-called ground truth); automatic tagging may replace humans in the loop and yields (mostly lower quality) machine-generated annotations, a computational process that generates *silver standard* data. Annotations mimic the human understanding process of unstructured data by requiring human annotators to strictly follow interpretation rules laid down in carefully crafted annotation guidelines. The outcome of annotation processes is quality-checked in terms of inter-annotator agreement metrics, whose scores indicate how close annotators adhere to annotation guidelines as language understanders; for comprehensive surveys on the role of corpora for machine learning and natural language processing (NLP), see Storks et al.^1^ and Paullada et al.,^2^ and for introductions to the organization of and methodology underlying annotation campaigns, see Lu^3^ and Ide and Pustejovsky.^4^ Annotated corpora are typically built with specific purposes in mind, for example, down-stream applications, such as text classification, named entity recognition, or relation/event extraction. Consequently, they normally address only 1 specific target layer of (language) understanding rather than its whole multi-dimensional spectrum.

Over the years, corpora have turned into an indispensable prerequisite for NLP since they serve 2 purposes. First, they provide the input for *machine learning* algorithms to learn structural and content properties from unstructured data. Second, annotated corpora constitute a common ground for evaluation experiments to measure the quality of systems operating on unstructured data in terms of (community-consensual) *benchmarks*. Hence, well-designed, reasonably sized, and publicly shared corpora are the foundation for the *reproducibility* of research results in that they allow the solid comparison of different types of language models, different sets of (hyper)parameters within the same model family, their effect on the outcomes of down-stream tasks, alternative system architectures, etc.

The dire need for specialized *clinical* corpora arises from the fact that medicine, as many other sciences, has established a highly diversified sublanguage on its own, diverging strongly from other scientific disciplines beyond the life sciences and, in particular, common language use patterns in everyday verbal communication.^5^^,^^6^ Even worse, clinical language is not homogeneous but splits into numerous subdomains and text genres,^7–9^ also differing from each other in many ways. Therefore, the utility of a given clinical/medical corpus must be carefully assessed in the light of various descriptive dimensions:

- Medical *subdomains* are often incompatible at the terminological level and follow different reporting standards. Consequently, documents from oncology are different from cardiology or radiology, and vice versa, both in terms of document structure and the verbalization of contents. This raises the issue whether a multitude of homogeneous *subdomain corpora* have to be supplied as an adequate pool for model training or, when such a large spectrum of subdomain corpora is lacking, whether models trained on, say, oncology data lead to poor(er) cardiology or radiology models, and vice versa.
  Liang et al.,^10^ eg, report on domain transfer learning experiments where PubMed-based generic medical language models (derived from medical journal abstracts) are applied to oncology data (the Bronco corpus)^11^ and nephrology data (the Ex4CDS corpus),^12^ respectively. Their results yield preliminary evidence that much of the enormous variance in the classification results can be attributed to the semantic alignment of (merged) named entity types to harmonize the corpora involved. In a follow-up study,^13^ the authors tackle this problem of semantic diversity by proposing a multi-layered semantic annotation scheme.
- Similar discrepancies can be observed for different clinical *text genres.* Their diversity is truly amazing. A *de facto* standard for the categorization of clinical documents in Germany, *Klinische Dokumentenklassen-Liste*, distinguishes more than 400 different genre types (https://simplifier.net/kdl/kdl-cs-2025) (We owe this information to Frank Meineke (personal communication).). Discharge summaries, for instance, differ significantly from pathology reports, radiology reports, operative reports, or nursing notes, both in terms of document structure and the verbalization of contents. Again, the question pops up whether homogeneous *genre-specific corpora* are needed for model training or, put the other way around, whether models trained on, say, discharge summary data lead to poor(er) pathology or radiology report models, and vice versa.
- Another crucial source of variance relates to *site-specific documentation standards*. For instance, discharge summaries from clinic A may deviate from those produced in clinic B and C, both in terms of document structure and verbal realization. This raises the question whether homogeneous *site-specific corpora* have to be generated for proper model training (even for the same clinical domain and clinical report genre) or, phrased alternatively, whether models trained on discharge summaries from clinic A are valid, at all, for discharge summaries from clinic B or C, and vice versa.
  Böhringer et al.^14^ conducted experiments to automatically infer ICD-10 codes in ophthalmologic departments of 3 different German hospitals and found that common eye disorders were mostly accurately classified by a language model trained in one of these hospitals and rolled out in the other 2 hospitals, whereas others, rare diseases in particular, varied considerably in classification accuracy. The authors also noted diverging local terminological standards and reporting habits, for example, the use of uncommon abbreviations that only make sense and are only understood in the local hospital environment. Such local “dialects” add an additional level of complexity to corpus-building initiatives that are hard to cope with.

Perhaps the most problematic issue with clinical/medical corpora is tied to the quest for prioritizing individual data privacy and security over distributability, which leads to extremely high hurdles, if not a non-negotiable blockade, to make such corpora publicly available. The underlying ethical concerns^15^ have been translated into legal protection regulations worldwide. These are intended to avoid individual patients’ re-identification once clinical documents leave safeguarded, hospital-internal data spaces, such as the patients’ Electronic Health Record (EHR). Criteria deserving such protection efforts have been spelled out most explicitly in the US HIPAA legislation act (https://www.hhs.gov/hipaa/index.html) and cover 18 privacy-sensitive attributes, so-called *Personally Identifiable Information* (PII; https://www.directives.doe.gov/terms_definitions/personally-identifiable-information-pii), which carry information about the patients’ and other clinical actors’ identity (see, eg, Table 2 in Seuss et al.^16^) HIPAA’s safe harbor rules require that such data items be neutralized by de-identification processes prior to allowing use by or disclosure to clinic-external individuals or institutions. Under these provisions, data distribution allowance usually requires signing a *Data Use Agreement* (DUA) between data owners (typically, a hospital) and external data users, which spells out detailed protective conditions for data storage and use and access at external sites. In Europe, the conditions of the *General Data Protection Regulation* (GDPR; https://gdpr.eu/) and national Germany data protection laws (eg, *“Gesundheitsdatennutzungsgesetz”* [GDNG; For a detailed discussion of German data protection regulations pertaining to clinical corpus distribution, see Lohr et al.^17^ [section 3]]) are less explicit in that they lack a comparable list of PII attributes. Instead, they require the explicit informed consent of data subjects for any external use. These requirements have the following implications:

- Clinical corpora may, under no circumstances, be made publicly available without complete and certified de-identification of PIIs. Certification and clearance are usually administered by the ethical board of the local hospital the data originate from.
- The features or attributes to be identified are explicitly enumerated for the US clinical NLP community (HIPAA’s PIIs). Such a clarification is missing in European law (GDPR) and national German law (GDNG). GDPR posits that data subjects have to express explicit consent that their de-identified data can be used for subsequent information processing; German law vaguely states that de-identified data can only be made publicly available when privacy can be broken with “unreasonable efforts” (what unreasonable efforts really are is not spelled out).
- The allowance for (fully de-identified) clinical corpora to be publicly distributed is always bound to the consent of the ethics board of the local hospital the corpus emerged from. This decision is based on verified adherence to the current legal data security ecosystem in Germany, as well as local hospital rules and best practices. Clinical administrations in Germany are extremely cautious to avoid potential juridical measures against data clearance and thus usually block corpus distribution.

Given this legal frame of reference, only very few German-language clinical corpora have been released for public use up until now. Consequently, the clinical NLP community in Germany has made immense efforts to replace real clinical corpora by reasonable substitutes or domain proxies. All these efforts are documented in detail in the Supplementary Material section of this article and will be summarized in the Results section.

## Objective

This review sheds light on corpus developments in the clinical and, more broadly, medical domain for the German language (spoken primarily in Germany, Austria, and parts of Switzerland by roughly 100 million native speakers). We will report on various real clinical corpora, almost all of them locked in safeguarded clinical data silos. Due to legal privacy protection regulations in Germany, clinic-external distribution of these corpora is usually forbidden, even after strict HIPAA-style de-identification, so that they remain inaccessible to the wider (clinical/medical) NLP community. Such rigid access restrictions violate established routines in NLP R&D workflows in which the (re-)usability of corpora is common practice for training and evaluating language models. Corpus developers have thus investigated several alternatives to bypass this data bottleneck. Hence, we will also review these potential substitutes for real clinical corpora in depth (for alternative surveys of German clinical corpora, see Starlinger et al.^18^ and Zesch and Bewersdorff^19^).

This review targets the following objectives:

- We provide a comprehensive survey of *German-language* corpora in the *clinical* domain and complement this narrow view by corpora with a wider *medical* scope.
- The corpora included in this review deal with *written* verbal data only. As far as multi-media data (eg, images in radiology reports) are concerned, only the written portion is dealt with. Speech corpora with spoken language as primary verbal data (eg, audio recordings of doctor–patient conversations) and any other modality complementing language behavior (visual information via deictic pointing gestures, body movements, facial expressions, etc.) will be excluded from this survey.
- We cover (hopefully) all corpora that have been published under peer review policy in the past quarter of a century, namely from *2000 until December 2024*.
- Abstracting away from the specifics of the individual corpora we survey, we introduce a generic template, we call *corpus card*, to guide future corpus descriptions (see the Supplementary Appendix, Table A1). This recommendation is language-independent and may be useful, in general, for the international medical informatics community to promote higher data science standards for corpus documentation.

## Materials and methods

We followed the PRISM (Preferred Reporting Items for Systematic reviews and Meta-analyses) guidelines for surveying the field of German-language clinical/medical corpora.^20^

### Study identification

Since the topic of this review lies at the intersection of (clinical) medicine and NLP, we considered a medical bibliographic resource (PubMed^®^, which comprises more than 37 million citations for biomedical literature from the bibliographic database Medline) and an NLP-focused one (ACL Anthology, with up to 100 000 bibliographic units from the most authoritative institution in the field of NLP, the *Association for Computational Linguistics*). As a third resource, we took Google Scholar (whose focus is on thematically unconstrained scholarly publications). Finally, the author’s own bibliographic database, ABib (with more than 66 000 bibliographic units covering [biomedical] NLP publications), was searched as well. The following queries were evaluated on August 24, 2024, on all 4 bibliographic databases (in addition, we conducted a final search on ABib on January 15, 2025, to collect the latest publications from 2024):

**PubMed**
  Query: (German) AND (text OR document) AND (corpus)
  Hits: 89
**ACL Anthology**
  Query: (German) AND (clinical OR medical) AND (corpus)
  Hits: 5510 (ordered by relevance)
**Google Scholar**
  Query: (German) AND (clinical OR medical) AND (corpus)
  Hits: ∼ 443 000 (ordered by relevance)
**ABib**
  Query: (language: German) AND (domain: medicine OR domain: clinic) AND (text corpus)
  Hits: 70 (+3) = 73

**Figure 1.**
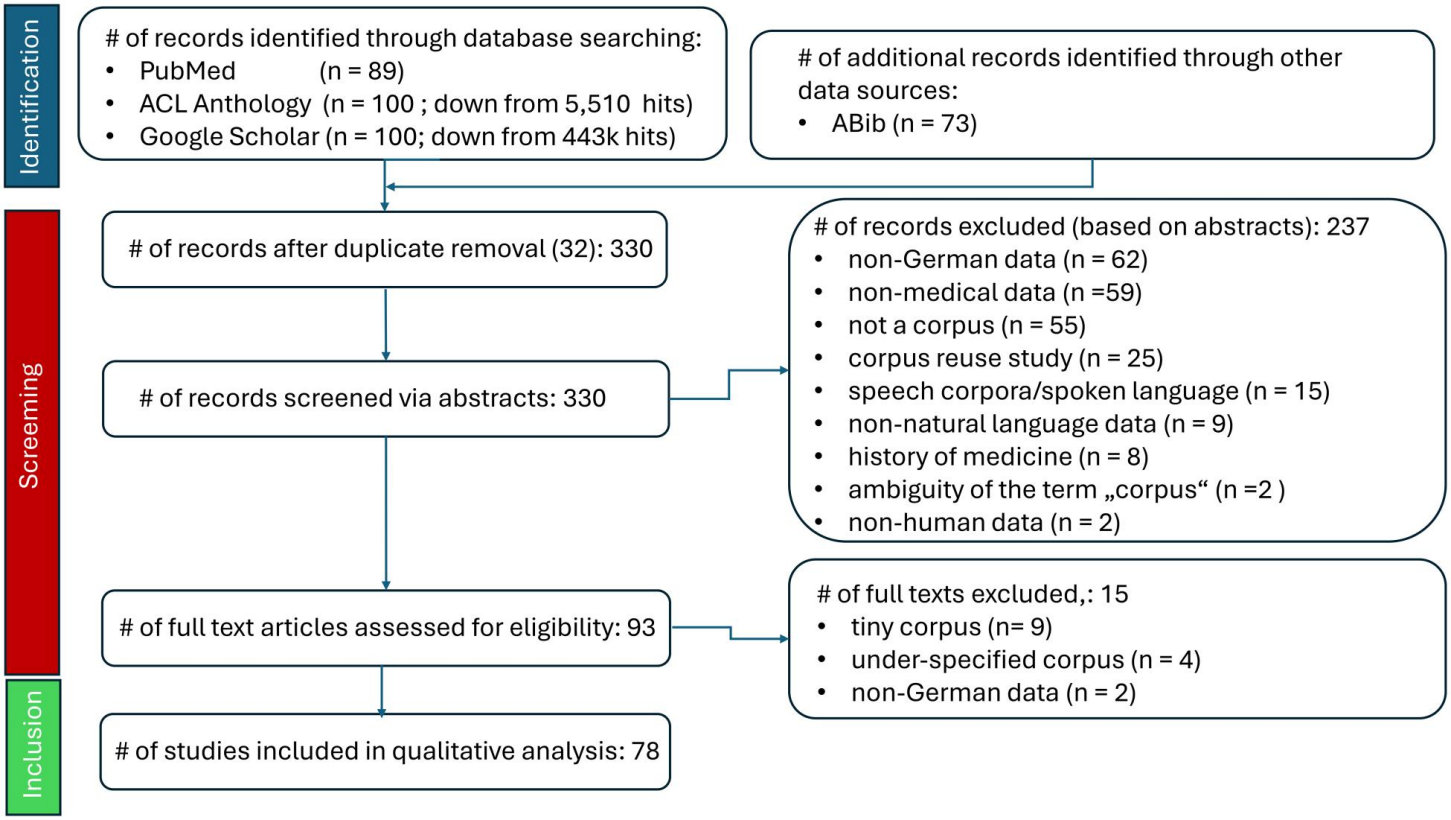
PRISM flowchart.

**Figure 2.**
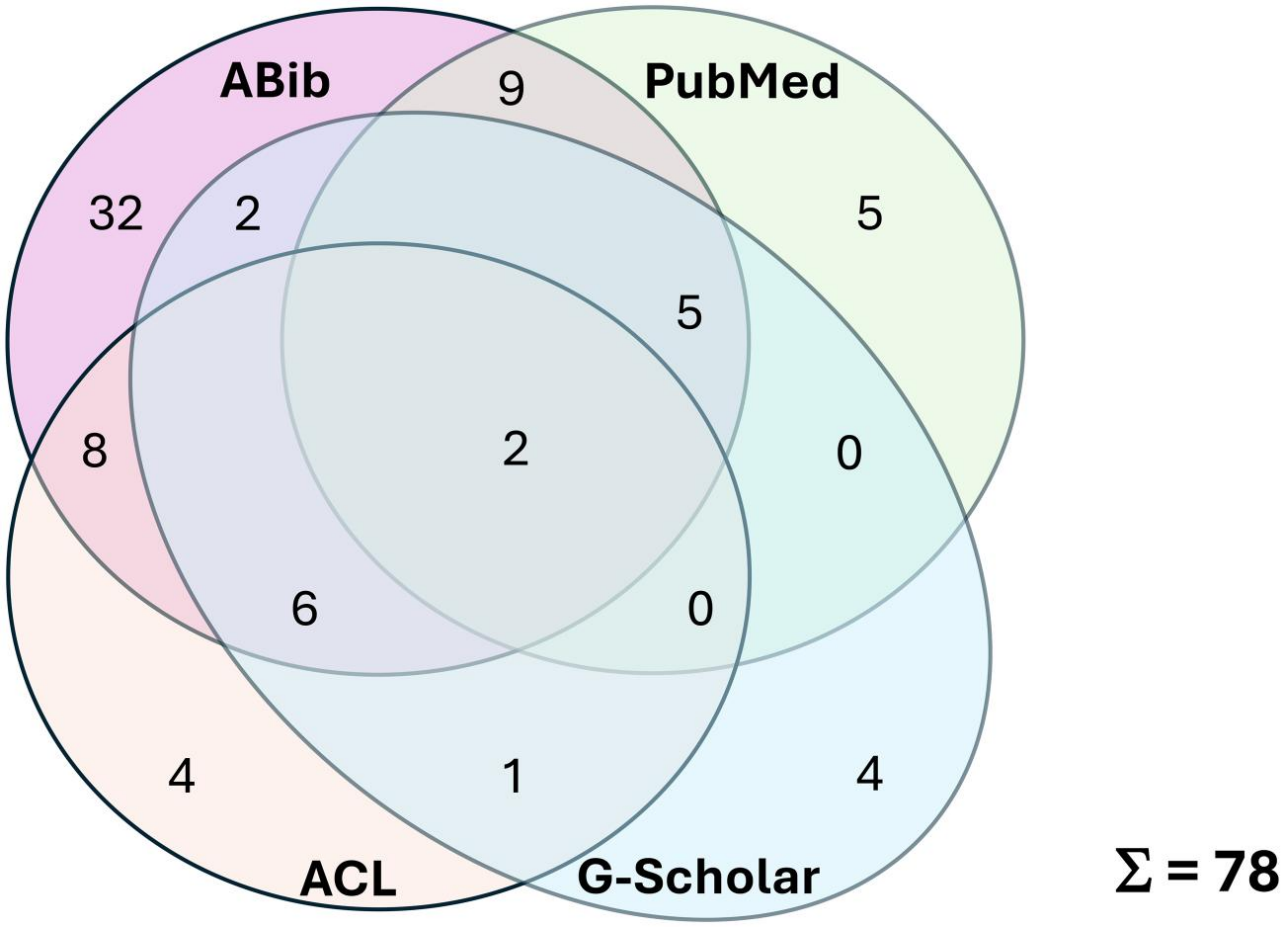
Distribution and overlap of relevant hits.

All hits were checked for PubMed (89) and ABib (73), whereas only the first 100 hits could be screened for ACL (the list was truncated after 100 hits by the search engine and could not be expanded) and Google (to mimic the procedure for ACL). The PRISM flowchart for the document selection process is depicted in Figure 1, while the distribution of all relevant articles and their overlaps for the 4 different search engines are displayed in Figure 2.

### Eligibility criteria

Only German-language clinical/medical corpora were eligible for this review; mixed-language corpora (eg, parallel corpora) were included if they contained a significant German portion (see criteria below). Publications that reused already existing corpora for down-stream applications were excluded, as well as corpora featuring *spoken* language, that is, audio data, whereas *written* chats, blogs, and tweets from social media channels or *written* doctor–patient conversations were included. Tiny corpora with less than 100 documents or less than 10 000 tokens were discarded (unless they are publicly shareable), as well as corpora portraying the history of medicine. Overly under-documented corpora lacking fundamental descriptive data (eg, number of documents or tokens) were also eliminated. We focused on human medicine only. The 4 independent searches yielded 362 hits altogether from which 78 were considered relevant and, thus, form the basis for this review (The spreadsheet for the relevance assessments of all 362 publications identified by the literature search is available from the author on demand.).

## Results

The following presentation of results is based on the division of corpus descriptions into 5 tables that can be found in the Supplementary Material (see Tables S2-S6). We distinguish between 3 types of clinical corpora (namely, real, translated, and synthetic ones) and 2 types of non-clinical medical corpora as domain proxies (mainly built from scholarly medical publications on the one hand and social media data and encyclopedic articles on the other hand). For all 5 categories of corpora, we distinguish between:

- the number of *publications* in which the individual corpora are described per category,
- the number of *distinct* (or *document-unique*) corpora per category, that is, ones with zero intersection of their document sets, or, alternatively, where different versions of the same corpus are genealogically aligned (this criterion merges identical document sets or sets of documents where one corpus is a superset of another one), and
- the number of *annotation-unique* corpora per category, that is, ones to which different types of metadata have been assigned (corpora lacking any metadata are excluded).

A summary table of all German-language clinical/medical corpora in which these three distinctions will be made concrete is provided in Table 1.

**Table 1.** Summary of German-language clinical/medical corpora.

Corpus type	Different corpus versions	Document-unique corpora	Annotation-unique corpora
Clinical—real	FraMed^21^ Müller-07^22^ Spat-08^23^ Kreuzthaler-11^24^ Fette-12^25^ Bretschneider-14^26^ Bretschneider-13^27^ Toepfer-15^28^ Lohr-16^29^ Löpprich-16^30^ Roller-16^31^ Roller-20^32^ Roller-22^33^ Cotik-16^34^ Roller-18^35^ Kreuztaler-16^36^ Seuss-17^16^ Oleynik-17^37^ Krebs-17^38^ 3000PA 5.0^39^ 3000PA 1.0^40^ 3000PA 2.0^41^ 3000PA 3.0^42^ 3000PA 4.0^43^ Becker-19^44^ Cardio:DE^45^ CardioAnno^46^ Richter- Pechanski-19^47^ König-19^48^ Bressem-24^49^ Bressem-20^50^ Grundel-21^51^ Bronco^11^ MedCorpInn^52^ MedCorpInn_sub_^52^ Karbun^53^ Madan-22^54^ [Ex4CDS]^12^ Trienes-22^55^ Llorca-23^56^ GeMTeX^57^ Böhringer-24^14^ Idrissi-Yaghir-24^58^ RadQA^58^ DMP “HerzMobil”^59^ Plagwitz-24^60^ **Σ:** 46	FraMed^21^ Müller-07^22^ Spat-08^23^ Kreuzthaler-11^24^ Fette-12^25^ Bretschneider-14^26^ *SuperSet-of* Bretschneider-13^27^ Toepfer-15^28^ Lohr-16^29^ Löpprich-16^30^ Roller-16^31^ = Roller-20^32^ *SuperSet-of* Roller-22^33^ Cotik-16^34^ Roller-18^35^ Kreuztaler-16^36^ Seuss-17^16^ Oleynik-17^37^ Krebs-17^38^ 3000PA 5.0^39^ *SuperSet-of* 3000PA 1.0^40^ *SuperSet-of* 3000PA 2.0^41^ 3000PA 3.0^42^ 3000PA 4.0^43^ Becker-19^44^ Cardio:DE^45^ *SuperSet-of* CardioAnno^46^ *SuperSet-of* Richter- Pechanski-19^47^ König-19^48^ Bressem-24^49^ *SuperSet-of* Bressem-20^50^ Grundel-21^51^ Bronco^11^ MedCorpInn^52^ *SuperSet-of* MedCorpInn_sub_^52^ *SuperSet-of* Karbun^53^ Madan-22^54^ [Ex4CDS]^12^ Trienes-22^55^ Llorca-23^56^ GeMTeX^57^ Böhringer-24^14^ Idrissi-Yaghir-24^58^ RadQA^58^ DMP “HerzMobil”^59^ Plagwitz-24^60^ **Σ: 32**	FraMed^21^ – Spat-08^23^ Kreuzthaler-11^24^ Fette-12^25^ Bretschneider-14^26^ Bretschneider-13^27^ Toepfer-15^28^ Lohr-16^29^ Löpprich-16^30^ Roller-16^31^ Roller-20^32^ Roller-22^33^ Cotik-16^34^ Roller-18^35^ Kreuztaler-16^36^ Seuss-17^16^ – Krebs-17^38^ 3000PA 5.0^39^ 3000PA 1.0^40^ 3000PA 2.0^41^ 3000PA 3.0^42^ 3000PA 4.0^43^ Becker-19^44^ Cardio:DE^45^ CardioAnno^46^ Richter- Pechanski-19^47^ König-19^48^ Bressem-24^49^ Bressem-20^50^ Grundel-21^51^ Bronco^11^ – – – Madan-22^54^ [Ex4CDS]^12^ Trienes-22^55^ Llorca-23^56^ GeMTeX^57^ Böhringer-24^14^ – RadQA^58^ DMP “HerzMobil”^59^ Plagwitz-24^60^ **Σ: 40**
Clinical—translated	Becker-16^61^ N2c2-German 2.0^62^ N2c2-German 1.0^63^^,^^64^ Idrissi-Yaghir-24^58^ **Σ: 5**	Becker-16^61^ N2c2-German 2.0^62^ *SuperSet-of* N2c2-German 1.0^63^^,^^64^ Idrissi-Yaghir-24^58^ **Σ: 3**	Becker-16^61^ N2c2-German 2.0^62^ N2c2-German 1.0^63^^,^^64^ – **Σ: 3**
Clinical—synthetic	JSynCC 2.0^39^ JSynCC 1.0^65^ GraSCCo 1.0^66^ GraSCCo 2.0^39^ GraSCCo 3.0_PHI_^17^ Frei-23^67^ **Σ: 6**	JSynCC 2.0^39^ *SuperSet-of* JSynCC 1.0^65^ GraSCCo 1.0^66^ = GraSCCo 2.0^39^ = GraSCCo 3.0_PHI_^17^ Frei-23^67^ **Σ: 3**	JSynCC 2.0^39^ JSynCC 1.0^65^ – GraSCCo 2.0^39^ GraSCCo 3.0_PHI_^17^ Frei-23^67^ **Σ: 5**
Close domain proxies	Brown-02^68^ MuchMore^69^ Springer-Link^70^ Springer^71^ MedTitle^71^ FraMed^21^ Morin-12^72^ Mantra [Silver]^73^ Mantra GSC^74^ HimL 1.0^75^ EFSG-UVigoMED^76^ Villena-20^77^ GGPOnc 2.0^78^ GGPOnc 1.0^79^ BTC^33^ ChaDL^80^ Bressem-24^49^ Idrissi-Yaghir^58^ **Σ: 18**	Brown-02^68^ MuchMore^69^ Springer-Link^70^ Springer^71^ MedTitle^71^ FraMed^21^ Morin-12^72^ Mantra [Silver]^73^ *SuperSet-of* Mantra GSC^74^ HimL 1.0^75^ EFSG-UVigoMED^76^ Villena-20^77^ GGPOnc 2.0^78^ *SuperSet-of* GGPOnc 1.0^79^ BTC^33^ ChaDL^80^ Bressem-24^49^ Idrissi-Yaghir^58^ **Σ: 16**	– MuchMore^69^ Springer-Link^70^ – – FraMed^21^ – Mantra [Silver]^73^ Mantra GSC^74^ – EFSG-UVigoMED^76^ – GGPOnc 2.0^78^ GGPOnc 1.0^79^ – – – – **Σ: 8**
Distant domain proxies	FraMed^21^ Lohr-16^29^ ML–UVigoMED^76^ WikiSection^81^ TLC-Med1^82^ RSS^83^ Beck-21^84^ Fang-Covid^85^ Lifeline 1.0^86^ BTC^33^ ChaDL^80^ Bressem-24^49^ Lifeline 2.0^87^ Heinrich-24^88^ HealthFC^89^ Pedrini-24^90^ Frei-24^91^ **Σ: 17**	FraMed^21^ Lohr-16^29^ ML–UVigoMED^76^ WikiSection^81^ TLC-Med1^82^ RSS^83^ Beck-21^84^ Fang-Covid^85^ Lifeline 1.0^86^ BTC^33^ ChaDL^80^ Bressem-24^49^ Lifeline 2.0^87^ Heinrich-24^88^ HealthFC^89^ Pedrini-24^90^ Frei-24^91^ **Σ: 17**	FraMed^21^ Lohr-16^29^ ML–UVigoMED^76^ WikiSection^81^ TLC-Med1^82^ RSS^83^ Beck-21^84^ Fang-Covid^85^ Lifeline 1.0^86^ – – – Lifeline 2.0^87^ Heinrich-24^88^ HealthFC^89^ – Frei-24^91^ **Σ: 13**

Overall	**Σ: 92**	**Σ: 71**	**Σ: 69**

### Clinical corpora

#### Real clinical corpora

Real clinical corpora are composed of original clinical reports or notes written by professional clinical staff who report about individual patients during their hospital stay. We found 46 publications for such corpora, from which 32 are distinct (document-unique), whereas 40 corpora are annotation-unique, that is, annotated with different types of metadata. Table S2 in the Supplementary Material section gives a detailed overview of these 46 corpora.

Clinical corpus construction efforts for the German language started in 2004 with FraMed.^21^ This corpus is medium-sized (100k tokens), annotated with low-level linguistic information only, and (due to the inclusion of clinical and copyrighted textbook material) non-sharable as a dataset. Language models for sentence and token splitting as well as part-of-speech tagging were made publicly available in the JCoRe model release 10 years later.^92^^,^^93^ From 2007 to 2016, various clinical corpora were developed as a by-product of application-focused studies, with Müller-07,^22^ Kreuzthaler-11,^24^ and Bretschneider-14,^26^ constituting, at that time, quantitatively outstanding datasets (roughly 30 000 documents [no token count], 3500 documents, 84k tokens, and 2700 documents, 347k tokens, respectively); Müller-07 and Kreuzthaler-11 come without any medically relevant metadata, whereas Bretschneider-14 has 148k tokens semantically annotated with domain-specific RadLex terms for radiology reports.

Around 2015, several new tendencies can be observed for corpus building in the German-language clinical NLP community. First, corpora, once created, undergo continuous quantitative augmentation, qualitative curation, and, in general, profit from iterative refinement in follow-up studies. Furthermore, the annotations feature fine-grained semantic information in terms of clinically relevant named entity and semantic relation types, as well as linguistic information covering, eg, negation and uncertainty signals. A typical example of this move are the activities of the Roller group,^31–35^ who developed a homogeneous corpus of discharge summaries in the nephrology domain (about 1725 [1360] documents, with some 158k [111k] tokens). It excels in the richest semantic type repertoire up until now (around 46k named entity annotations for 17 types and 17k relation annotations for 9 types in the latest, slightly downsized release).^33^ For the first time ever, also a DUA-based access option for pre-trained information extraction models is provided. The approach taken by the 3000PA team^39–43^ is perhaps even more ambitious, since their work (based on more than 6600 clinical documents, mainly discharge summaries, from 3 different national university hospitals, with 7.3 million tokens)^39^ aims at the broad coverage of very diverse annotation layers ranging from medication information (1 entity type, 5 relations),^40^ 18 section heading types,^41^ 13 PII entity types,^42^ 3 medical named entity types (Symptom, Finding, Diagnosis),^43^ various semantic relations, as well as factuality and temporal information.^39^ All this accumulates in slightly more than 2 million annotation items in the final release,^39^ a metadata resource unmatched in quantity and breadth so far. Work on Cardio:DE (formerly named CardioAnno)^46^ features 500 clinical reports (993k tokens) in the cardiology domain,^45^ with 12 cardiovascular entity types (1.6k annotation units),^46^ 14 section heading types (116.9k annotation units), 2 named entity types for medication, and 7 relation types (26.6k annotation units).^45^ Unlike all the previously built corpora, Cardio:DE is publicly available on a DUA basis. Finally, the work of Bressem et al.^50^ features 6000 radiology reports (estimated 850k tokens), with annotations relating to 9 Finding types (15k annotation units), the presence/absence of 4 pathologies, and 4 different types of therapy devices.^49^ The radiology core of this corpus remains locked, yet the RadBert language model for extracting Finding types,^50^ as well as pretrained model weights for the medBert language model and radiology benchmarks, can be distributed.^49^ These studies, fully compliant with mainstream non-medical NLP, also mark a fundamental change of the role of corpora in clinical NLP—originally conceived as a side issue of application-centered research their design and realization now has become a respected research theme on its own.

Judging the potential value of clinical corpora merely in terms of the number of documents or tokens is only a weak quality indicator. For instance, the largest corpora in terms of the number of documents, Idrissi-Yaghir-24,^58^ with slightly more than 25 000k documents, MedCorpInn,^52^ with 5000k documents, Grundel-21,^51^ with 40.5k documents, and Oleynik-17,^37^ with 30k documents, all suffer from the lack of any clinically relevant metadata (Grundel-21 inherits gold standard data from the structured part of the parallel EHR from which the documents were extracted). Within this group of very large corpora, only DMP “HerzMobil”,^59^ with roughly 36k documents, carries medically relevant semantic annotations, yet these are automatically generated and thus form a silver standard corpus. Also, due to the nature of different clinical document genres (eg, discharge summaries being much longer than clinical notes), the number of tokens does not necessarily increase with the number of documents. As a more expressive yardstick for content-based corpus assessment, one might consider the numbers of semantically rich, medically relevant annotations. On that scale, the following corpora are top-ranked:

- 3000PA 5.0,^39^ with 6600 documents (7300k tokens), composed of discharge summaries from multiple clinical sites, with 2093k multi-level annotation units,
- Cardio:DE,^45^ with 500 documents (993k tokens), composed of clinical reports from the cardiology domain, with 143.5k named entity and relation annotations,
- Roller-20,^32^ with 1725 documents (158k tokens), composed of discharge summaries from the nephrology domain, with 77.4k named entity and relation annotations.

Sheer numbers relating to documents, tokens, and medical metadata are but one side of the coin for corpus assessment. On the flipside, their accessibility to a wider R&D community is even more important for data-driven scientific progress. Here comes the bad news—out of 32 document-unique corpora, only 5 are externally accessible at all, yet with different clearance policies. A historical breakthrough was achieved with Bronco,^11^ a collection of 200 discharge summaries (90k tokens), with annotations for section headings and 3 named entity types, namely, Diagnosis, Treatment, and Medication, plus their grounding in ICD-10, OPS, and ATC terminologies, respectively. Unfortunately, this pioneering work, formally accessible via DUA, is devalued by the fact that the 11k sentences in this corpus were arbitrarily shuffled (for increased privacy protection) so that the entire document structure has been intentionally spoiled. Hence, Cardio:DE composed of 500 clinical reports from the cardiology domain can be considered the first and only German-language clinical corpus whose structure is left intact (after de-identification) and whose accessibility is implemented via DUA as well.^45^ Since Bronco and Cardio:DE follow a formalized DUA-based clearance policy, they strictly adhere to internationally established distribution standards for privacy-sensitive medical corpora.

Böhringer-24, composed of 300 ophthalmologic physicians’ letters from 3 different hospitals and annotated with 2800 diagnoses from ICD, is the third in this line but raises concerns because potential clearance requires informal private negotiations, which may end up in a formal DUA if permission is finally granted.^14^ Ongoing work on GeMTeX,^57^ a currently active major national corpus-building initiative, targets an even larger (>150k documents) and more heterogeneous collection of clinical report types covering 4 medical areas (cardiology, pathology, pharmacy, and neurology) from 6 different national clinical sites. This corpus is still *in statu nascendi* and thus currently not ready for distribution in a routine manner. Interestingly and for the first time ever in Germany, all documents entering GeMTeX require GDPR-conformant “informed consent,” that is, the explicit agreement of patients that their clinical documents can be used (in de-identified form) for research purposes; however, potential clearance will still require some sort of DUA. Whereas these 4 corpora all feature standard clinical text genres (mostly discharge summaries), the fifth one, Ex4CDS,^12^ stands out as a non-standard clinical corpus. It is composed of 720 physicians’ justifications supporting their estimated likelihood of future possible negative patient outcomes after kidney transplantations. This genre heavily drifts away from standard reporting formats we see in common clinical reports and notes, and, thus, might be of minor relevance compared with Cardio:DE, Bronco, GeMTeX, and Böhringer-24. Summarizing, only 15% (5) of all document-unique real German-language clinical corpora (32) out of a total of 46 publications are open for the scientific community under most optimistic assumptions, yet only 6% (2) are currently ready for distribution under a standardized formal DUA protocol (comparable, eg, with Mimic distribution standards; https://physionet.org/content/mimiciii/1.4/).

Once more and more single clinical/medical corpora become publicly available, potential synergies arising from their combination can be explored. Llorca et al.^56^ describe such an approach for 4 corpora (Bronco, Cardio:DE, GGPOnc 2.0, and GraSCCo; the latter 2 will be introduced below) using the BigBIO framework for (meta)data harmonization.^94^

It is also worth noting that several attempts have been made to distribute language *models* (rather than the original non-distributable clinical raw text *data*) that were derived from classified local clinical resources (see FraMed,^21^ Bressem-20,^50^ Roller-20,^32^ Roller-22,^33^ and Bressem-24^49^) Still these options open unexplored legal territory and face problems on their own (we will touch upon the entailed data security issues below).

#### Translated real clinical corpora

Translated real clinical corpora are derived from real clinical reports and notes routinely written by professional clinical staff yet have been automatically translated from (easier to get) US-American English sources to German. We found 5 publications for such corpora, from which 3 are document-unique and, also, 3 corpora are annotation-unique (actually, only 2 corpora, since one of them—N2c2-German 2.0—differs only in terms of the number of annotated items in its most current version, not type-wise). Table S3 in the Supplementary Material section gives a detailed overview of these 5 corpora.

Becker-16^61^ relies on ShARe/CLEF eHealth 2013 Shared Task 1 resources^95^ that reused Mimic-II data, whereas the n2c2-German corpus^62–64^ builds on n2c2 2018 Shared Task Track 2 data^96^ that exploited Mimic-III data. Their size is moderate (200^61^ and 400 documents,^62^ respectively, the latter with almost 370k tokens). Discharge summaries prevail, and the annotations relate to named entity (Disorders, Drugs) and relation extraction (Medication/Adverse Drug Events) tasks, with up to 63.4k annotation units. Idrissi-Yaghir-24^58^ makes use of a much larger segment of Mimic-III, with 695 000k tokens after translation into German (yet without specification of the basic number of documents and without any metadata). Not as a surprise, all these corpora are publicly accessible (they inherit Mimic’s liberal DUA policy) and both versions of n2c2-German also offer a free named entity recognition model.

There are 3 issues with this approach. First, the quality of the automatic translation needs thorough human review by medical experts. Second, the proper alignment of the metadata must be manually validated, since begin/end positions of metadata are likely to change from English to German documents. Beyond these translation-focused issues, at a more “cultural” level, the writing style of American doctors tends to deviate from that of German ones reflecting a different reporting culture embedded in incompatible health care eco-systems. Initial experimental results on the effects of translated English documents for German clinical language models are reported by Idrissi-Yaghir et al.,^58^ although clinical data (from Mimic-III) and non-clinical ones (from PubMed) are indistinguishably intertwined in their experimental design.

#### Synthetic clinical corpora

Synthetic clinical corpora feature invented clinical reports and notes that look like those written by professional clinical staff in terms of genre, style, and terminology but describe entirely fictitious patients and artificially constructed or massively altered medical cases. Synthetic documents are typically authored by medical experts of the same professional caliber as those authoring real ones, either by *manually writing* them from scratch or by *manually re-writing* original exemplars. With the increasing power of large language models (LLMs) rooted in the deep learning (DL) paradigm, the advent of ChatGPT^97^^,^^98^ in particular, the *automatic generation* or *automatic paraphrasing* of clinical documents has become a feasible machine alternative based on prompts (instructions issued by human users that control and help tailor LLM system output). We found 6 publications for such corpora, from which 3 are document-unique and 5 are annotation-unique. Table S4 in the Supplementary Material section gives a detailed overview of these 6 corpora.

JSynCC 1.0^65^ was the first of its kind for the German clinical language and consists of 400 operative reports and 470 case reports/descriptions extracted from e-book versions of introductory textbooks for medical students. Since this corpus cannot be shared directly due to Intellectual Property Rights held by the publishers, the developers bypassed this restriction by distributing the code to reliably re-create JSynCC copies at any other physical site (including selected metadata). As a prerequisite, the e-books incorporated in JSynCC need to be licensed by that local institution. In the meantime, JSynCC 2.0^39^ contains 343k annotation units covering various named entities, such as Findings, Diagnoses, Procedures, and PII.

GraSCCo can be considered a true representative of the re-writing paradigm. Despite its tiny size (63 documents, 44k tokens only), the original version, GraSCCo 1.0,^66^ has developed into GraSCCo 2.0^39^ with different kinds of named entities, semantic relations, temporal relations, certainty, and negation tags, amounting to nearly 180k annotation units altogether. It is publicly accessible without any restrictions, and its most recent version, GraSCCo 3.0_PHI,_^17^ also incorporates 1.4k PII annotation units. GraSCCo is based on real discharge summaries and web-crawled clinical documents that were massively linguistically edited, with iterative changes at the lexical, syntactic and semantic level. Furthermore, medical noise (new data items, new attribute-value sets, etc.) was intentionally injected for reasons of camouflage so that re-identification of individual patients seems virtually impossible.

As to *automatic text generation* based on LLMs, Frei-23^67^ uses a prompt-based approach to generate (roughly 10k) new single sentences (*not* full-fledged documents!), which amount to slightly more than 120k tokens. An automatically generated silver standard includes 3 named entity types (Medication, Dose, and Diagnosis) comprising roughly 23k silver annotation units. As with GraSCCo, Frei-23 is publicly available without any constraints.

The motivation for and general advantage of synthetic corpora is that they circumvent the data protection problem as virtual patients and artificial cases are constructed and verbalized. However, one may question whether synthetic documents, either written by medical experts or DL engines, sufficiently correspond with much more heterogeneous real ones and thus can really replace them without substantial analytic biases. For instance, case descriptions, in particular those published in textbooks, deviate from authentic clinical reports in terms of a more narrative, often verbose style and non-expert language use whereas clinical case reports resemble scholarly articles, i.e., unlike clinical reports they use well-formed grammatical language and standardized expert terminology.

Concerning synthetic datasets, Şerbetçi and Leser^99^ report preliminary evidence that models trained on the synthetic data from Frei-23 do not transfer well to authentic clinical data from Bronco and Cardio: DE. A bunch of alternative experiments considering other natural languages, however, consistently reveal contrary evidence. For instance, Kweon et al.^100^ demonstrate that synthetic English clinical notes (i.e., coherent long texts) generated from an LLM can serve as viable substitutes for real ones by comparing models trained on these synthetic data with ones trained on real clinical data (synthetic data showing only a slight decrease in performance relative to real data). Similar encouraging results for synthetic document sets (again, showing slight performance drops when models are trained on synthetic data and tested on real data) are reported by Hiebel et al.^101^ for French named entity recognition and Brekke et al.^102^ for the extraction of family history information from Norwegian clinical notes. Li et al.^103^ and Ive et al.^104^ also report on English data where the synthetic corpus shows a high degree of usability as a substitute for real clinical corpora. The negative result for German might presumably be traced to the complete lack of document structure in the collection of single clinical sentences provided in Frei-23. Hence, one may summarize that ample empirical evidence has been gathered in favor of synthetic clinical datasets as valid substitutes for real ones with low, almost negligible penalty costs, though not for German, so far. Despite these good news, there is a caveat. Privacy attack experiments also revealed that reverse engineering from embeddings allows read-outs of sensitive factual data (eg, PII) from LLMs via training data extraction attacks^105^^,^^106^—even in their de-identified form via a similarity search attack^107^—and therefore bears an unwanted potential for data privacy breach.

#### Close domain proxies: pseudo-clinical corpora

*Domain proxies* for clinical corpora are collections of documents that deal with medical topics but differ from clinical reports mostly in terms of style and genre. We further refine this category in this subsection as *close* domain proxies when clinical topics are dealt with from a *scholarly* perspective at an *expert* medical level; they constitute the class of *pseudo-clinical corpora*. Perhaps the largest source of such documents is housed in PubMed-style bibliographic databases or publishers’ Web portals hosting titles, abstracts, or full texts of academic journal articles. Additional material comes from medical PhD theses, clinical guidelines, clinical trial reports, drug labels, or patent claims. We found 18 publications for such corpora from which 16 are document-unique and only 8 are annotation-unique. Table S5 in the Supplementary Material section gives a detailed overview of these 18 corpora.

By far the largest group, composed of 14 corpora (Brown-02,^68^ MuchMore,^69^ SpringerLink,^70^ Springer + MedTitle,^71^ Morin-12,^72^ Mantra Silver + Mantra GSC,^73^^,^^74^ HimL 1.0,^75^ EFSG-UVigoMED,^76^ BTC,^33^ ChaDL,^80^ Idrissi-Yaghir-24,^58^ and Bressem-24^49^) makes a second-hand use of collections from bibliographic databases, such as PubMed/Medline or Livivo, or commercial publishers’ websites. Eight of them are parallel/comparable multilingual corpora, with German as one of the featured languages (Brown-02, MuchMore, Springer and MedTitle, Morin-12, Mantra Silver + Mantra GSC, and EFSG-UVigoMED). These proxies typically excel in huge data volumes—Idrissi-Yaghir-24 offers the largest dataset with roughly 6m documents (about 1,7b tokens), followed by Mantra Silver with some 4.3m documents (more than 60m tokens), and HimL 1.0 with roughly 2.7m documents (slightly less than 60m tokens). Not surprisingly, these massive data volumes come at the price of lacking annotations. Whereas Idrissi-Yaghir-24 and HimL 1.0 contain no metadata at all, Mantra Silver introduced the notion of a *silver standard corpus*, that is, a huge number of automatically generated annotations as the result of harmonizing the contributions of ensembles of named entity taggers.

A second, much smaller group of corpora contains textual data from drug labels and patent claims (Mantra Silver + Mantra GSC, HimL 1.0, and ChaDL). The third one is constituted by GGPOnc, which consists of clinical guidelines for oncology.^78^^,^^79^ It not only stands out as a unique guideline corpus publicly available via DUA but is large-sized (about 10k text segments from the complete set of 30 German oncology guidelines, with roughly 1900k tokens) and excels in annotations with either 7 named entity types (GGPOnc 1.0)^79^ taken from the UMLS Semantic Groups (with around 73.8k annotation units) or 3 Snomed CT-anchored named entity types (GGPOnc 2.0)^78^ currently summing up to roughly 450k curated annotation units.^39^^,^^78^

Scholarly writing is fundamentally different from clinical writing—not only in terms of genre and style but also in terms of language use characteristics. Whereas scholarly articles mostly adhere to linguistic well-formedness, terminological canonicity, and definitional rigor, clinical reports abound with paragrammatical syntax, spelling errors, and local clinical jargon (exemplified by in-house abbreviations or acronyms) typical of language performance under high workload and, thus, heavy time pressure, as well as closed language community conventions. Perhaps the main difference, however, lies in their diverging communicative intention—whereas academic writing usually addresses the generalizability of observables (eg, the effect of a drug or a medical procedure within a patient cohort), clinical reports focus on individual patients only. Whether these considerations have a measurable impact on training or adapting language models remains an issue of further research.

#### Distant domain proxies: non-clinical medical corpora

*Distant* domain proxies for clinical corpora are sets of documents covering medical topics from a non-clinical perspective, targeting mainly non-expert comprehensibility, here referred to as *non-clinical medical corpora*. In this group, the genre-specific style of clinical reporting vanishes completely, although lexical adherence to medical terminology is sought for, though often at a layman level (eg, “*Blinddarmentzündung*” is preferred over “*Appendicitis*,” “*Blutvergiftung*” over “*Sepsis*”). We found 17 publications for such corpora from which all 17 are document-unique, whereas 13 are annotation-unique. Table S6 in the Supplementary Material section gives a detailed overview of these 17 corpora.

The dominant group of distant domain corpora is composed of 10 resources in which *social media* data are assembled, either incorporating medically focused chats extracted from general social media platforms, such as Twitter or Telegram,^84^^,^^85^^,^^88^ or from thematically specialized public health portals, for example, dealing with diabetes, obesity, drug misuse, adverse drug events, or depression. Though a layman language attitude prevails in this *dialogical* data, medical expert statements can be found here as well, particularly in public health portals, but rigorous medical expert jargon is typically avoided or, if it pops up, translated into more understandable layman language. Exemplars of social media medical corpora are TLC-Med1,^82^ which collects excerpts from the German Med1.de health portal, LifeLine,^86^^,^^87^ which contains threads thematically related to adverse drug reactions, BTC,^33^ Bressem-24,^49^ and HealthFC^89^ (a claim–evidence–verdict triple dataset for fact checking). The data volume varies a lot in this category—from half a million tokens in TLC-Med1, Beck-21, or HealthFC via 9m tokens in Bressem-24 up to 32m tokens in Fang-Covid.

A second class of distant domain corpora is formed by 5 resources composed of (*monological*) online *encyclopedic articles* dealing with medical topics as available, for example, from Wikipedia. Typical examples of this approach are WikiSection (basically a disease corpus),^81^ ChaDL,^80^ Bressem-24,^49^ or Frei-24.^91^ These are also high-volume datasets, with 2-4k documents (2-3m tokens), ChaDL with more than 20m tokens being the largest one.

Finally, perhaps the most distant, collections of general *newspaper/newswire articles* are assembled in corpora dealing with medical themes, such as Lohr-16,^29^ RSS,^83^ and Fang-Covid.^85^ These corpora are typically supersized, with (tens up to hundreds of) millions of tokens, yet without any metadata; Lohr-16 is top-ranked here reaching 126m tokens.

Not surprisingly, all these corpora are publicly available, although care should be taken when social media data are chosen, for example, from health consultation or disease community portals, where privacy issues easily pop up.^108^^,^^109^ Distant domain proxies are typically large-sized, with millions of tokens, but often lack expressive medical metadata (WikiSection, TLC-Med1, Beck-21, Lifeline 2.0, Heinrich-24, HealthFC, and Frei-24 being notable exceptions from this rule). Fundamental concerns may be raised whether these sources can reasonably be used, at all, as a substitute for clinical data due to heavily divergent genre, style, argumentation, and vocabulary patterns.

## Discussion

Corpora are an indispensable prerequisite for training, tuning, adapting, and evaluating (large) language models (Activities related to generating clinical German-language models that make use of many of the corpora introduced in this review are reported in, for example [^10^^,^  ^13^^,^^14^^,^  ^39^^,^  ^80^^,^  ^110–113^].). In the clinical domain, however, these resources are hard to get because of ethical concerns that have been translated into rigorous data protection laws worldwide. In Germany, for instance, at the time of this writing (January 2025) 27 non-distributable, yet often richly annotated clinical datasets are kept in closed local data silos inaccessible for clinic-external researchers. This constitutes not only an enormous waste of money and human resources but also a serious loss of medical opportunities for better diagnosis and treatment—not to mention the reduction of costs for the health care system. Fortunately, this (over-)protective siloing strategy is starting to become more permeable, as witnessed by the strictly *DUA*-formalized accessibility of the Cardio:DE^45^ and Bronco^11^ clinical report corpora. Three additional corpora may be counted as potential alternatives—Böhringer-24^14^ (though the access option rests on an informal distribution offer), GeMTeX^57^ (a corpus-building initiative just launched, the results of which will only be available in the course of 2025 but are fully compliant with EU regulations [GDPR] based on informed consent), and Ex4CDS,^12^ whose domain of discourse (risk justifications after kidney transplantations) is somewhat off-topic compared with standard clinical reports and notes.

Several researchers offer a bypass in that they do not distribute locked clinical *raw data* or associated *metadata* but rather allow the *language models* generated from these original data to be distributed. One caveat must be made—data privacy issues may pop up here since evidence has been reported that individual patients’ data can indeed be read out from the models’ representation structures and thus bear the danger of patient re-identification demanding further safety measures against hostile attacks.^105–107^

That said, we also looked at alternative corpus designs that have been investigated to escape from clinical text data sparsity. We organized these efforts in a taxonomy based on qualitative considerations. For *real* clinical corpora, we found 2 ways to circumvent data access restrictions. The first one is to pick up DUA-accessible English data and *translate* them automatically. The second strategy is to generate, manually or automatically, *synthetic* clinical reports with fictitious content.

As another alternative, we identified *domain proxies* for clinical reports. They deal with clinical or, more general, medical, topics written by medical experts or laymen, yet depart from standard clinical report writing in terms of genre, style, and terminology to a varying degree, though. The category of *close* domain proxies is constituted by pseudo-clinical documents, such as the whole range of scientific medical literature (abstracts and full texts from journals), therapy guidelines, clinical trial reports, drug labels/leaflets, or patent claims. Also, more *distant* domain proxies play a role here, namely those that deal with medical themes without clinical phrasing, because their target is a general, non-expert audience. This category is filled either by chats, threads, or tweets from generic social media channels or specialized health portals, or by encyclopedic articles from Wikipedia. Altogether (see Table 1), we identified 71 distinct, that is, document-unique, and 69 annotation-unique German-language corpora from 92 publications (Some corpora were assigned to more than one of the 5 categories. Therefore, this publication count (92) is higher than the number of relevant hits (78)).

Idrissi-Yaghir-24^58^ is currently by far the largest of all German-language medical corpora, with slightly more than 25m documents and 3.0b tokens from its clinical segment, plus the translated Mimic-III clinical segment (695m tokens), plus the translated PubMed segment (6000k abstracts with 1 700m tokens)—roundabout more than 31m documents with 5.4b tokens. The vast clinical portion of this corpus is used for in-house training of the clinical language model—a recent trend leading to hospital-specific language models without the need for de-identification and data sharing. Bressem-24^49^ is even more heterogeneous and the second-largest medical German-language corpus, a hybrid conglomerate of clinical reports, embedded public corpora (GGPOnc, GraSCCo), a PubMed subset, publisher-provided scientific papers, and medical PhD theses—overall, more than 4.7m documents (1.1b tokens).

Their sheer amount of tokens is truly impressive, but when it comes to the supply of clinically relevant metadata, other corpora deserve at least equal credit. On this dimension, we find:

- 3000PA 5.0,^39^ with 6600 documents (7.300k tokens) and 2093k multi-level annotation units, including section, named entity and relation annotations, as well as annotations involving temporality and factuality,
- Cardio:DE,^45^ with 500 documents (993k tokens) and 143.5k named entity and relation annotations,
- Roller-20,^32^ with 1725 documents (158k tokens) with 77.4k named entity and relation annotations.

Among these 3 corpora, Cardio:DE stands out as the only one that is accessible on a formalized DUA basis (together with the smaller and less richly annotated Bronco corpus).

Still, the taxonomy we introduced leaves open an important issue: How close/distant, in a metrical sense, are potential substitutes when compared with real clinical reports in terms of genre, style, jargon, and diction? This *stylometric* question should be complemented by a *functional* one: How good are these substitutes in terms of classification performance when compared to real clinical documents? Initial attempts at answering these emerging research questions have already been made. Modersohn et al.^66^ compared a synthetic clinical corpus (GraSCCo) with a real one (3000PA) by clustering syntactic and semantic features, whereas Lohr and Hahn^114^ developed DoPA Meter, a stylometric toolkit with more than 120 style metrics covering lexical, syntactic, and semantic expression layers, and ran it on synthetic as well as on close and distant domain proxies. However, a comprehensive functional comparison is still lacking, although first experiments have been reported for Cardio:DE, Bronco, GGPOnc 2.0, and GraSCCo by Llorca et al.^56^ and Şerbetçi and Leser.^99^ Stylometric analyses could highlight descriptive differences in terms of linguistic variance, whereas an experimental comparison of the (classification) performance of language models trained on real clinical corpora with ones trained on translated, synthetic, and proximal substitutes could lead to an empirically founded “cost model” for corpus substitution.

## Supplementary Material

ooaf024_Supplementary_Data

## Data Availability

The spreadsheet for the assessment of the 330 screened publications is available from the author upon request. The author will provide an incremental update service for the tables from this publication to reflect the on-going changes involving German-language clinical text corpora. Please, contact the author via e-mail.

## References

[1] Storks S , Gao Q, Chai JY. Recent advances in natural language inference: a survey of benchmarks, resources, and approaches. arXiv: 1904.01172 (v2). https://arxiv.org/pdf/1904.01172, 2020, preprint: not peer reviewed.

[2] Paullada A , Raji ID, Bender EM, et al Data and its (dis)contents: a survey of dataset development and use in machine learning research. Patterns. 2021;2:100336. 34820643 10.1016/j.patter.2021.100336 PMC8600147

[3] Lu X. Computational Methods for Corpus Annotation and Analysis. Springer; 2014.

[4] Ide NC , Pustejovsky JD, eds. Handbook of Linguistic Annotation. Springer; 2017.

[5] Campbell DA , Johnson SB. Comparing syntactic complexity in medical and non-medical corpora. In: *AMIA Annu Symp Proc 2001. A Medical Informatics Odyssey: Visions of the Future and Lessons from the Past*, Washington, DC, USA, November 3-7, 2001. Hanley & Belfus; 2001:90-94. PMC2243419 11825160

[6] Friedman C , Kra P, Rzhetsky A. Two biomedical sublanguages: a description based on the theories of Zellig Harris. J Biomed Inform. 2002;35:222-235. 12755517 10.1016/s1532-0464(03)00012-1

[7] Zeng QT , Redd D, Divita G, et al Characterizing clinical text and sublanguage: a case study of the VA clinical notes. J Health Med Inform. 2011;2011:S3.

[8] Patterson OV , Hurdle JF. Document clustering of clinical narratives: a systematic study of clinical sublanguages. In: *AMIA Annu Symp Proc 2011. Improving Health: Informatics and IT Changing the World*, Washington, DC, USA, October 22-26, 2011:1099-1107. PMC3243234 22195171

[9] Lysanets Y , Morokhovets H, Bieliaieva O. Stylistic features of case reports as a genre of medical discourse. J Med Case Rep. 2017;11:83. 28285584 10.1186/s13256-017-1247-x PMC5346841

[10] Liang S , Hartmann M, Sonntag D. Cross-domain German medical named entity recognition using a pre-trained language model and unified medical semantic types. In: *ClinicalNLP 2023—Proceedings of the 5th Workshop on Clinical Natural Language Processing @ ACL 2023*, Toronto, Ontario, Canada, July 14, 2023; 2023:259-271.

[11] Kittner M , Lamping M, Rieke DT, et al Annotation and initial evaluation of a large annotated German oncological corpus. JAMIA Open. 2021;4:ooab025. 33898938 10.1093/jamiaopen/ooab025 PMC8054032

[12] Roller R , Burchardt A, Feldhus N, et al An annotated corpus of textual explanations for clinical decision support. In: *LREC 2022—Proceedings of the 13th International Conference on Language Resources and Evaluation*, Marseille, France, June 20-25, 2022; 2022:2317-2326.

[13] Liang S , Profitlich H-J, Klass M, et al Building a German clinical named entity recognition system without in-domain training data. In: *ClinicalNLP 2024—Proceedings of the 6th Workshop on Clinical Natural Language Processing @ NAACL 2024*, Mexico City, Mexico, June 21, 2024; 2024:70-81.

[14] Böhringer D , Angelova P, Fuhrmann L, et al Automatic inference of ICD-10 codes from German ophthalmologic physicians’ letters using natural language processing. Sci Rep. 2024;14:9035. 38641674 10.1038/s41598-024-59926-3 PMC11031573

[15] Šuster S , Tulkens S, Daelemans WA. Short review of ethical challenges in clinical natural language processing. In: *Proceedings of the 1st ACL Workshop on Ethics in Natural Language Processing @ EACL 2017*, Valencia, Spain, April 4, 2017; 2017:80-87.

[16] Seuss H , Dankerl P, Ihle M, et al Semi-automated de-identification of German content sensitive reports for big data analytics. Rofo. 2017;189:661-671. 28335044 10.1055/s-0043-102939

[17] Lohr C , Matthies F, Faller J, et al De-identifying GraSCCo: a pilot study for the de-identification of the German Medical Text Project (GeMTeX) corpus. In: *German Medical Data Sciences 2024. Health—Thinking, Researching and Acting Together*. *Proceedings of the 69th Annual Meeting of the German Association of Medical Informatics, Biometry, and Epidemiology e.V. (GMDS) 2024*, Dresden, Germany, 8-13 September 2024. 2024:171-179 (*Stud Health Technol Inform*, 317). 10.3233/SHTI240853 39234720

[18] Starlinger J , Kittner M, Blankenstein O, et al How to improve information extraction from German medical notes. it—Information Technology. 2016;58:1-8.

[19] Zesch T , Bewersdorff J. German medical natural language processing: a data-centric survey. In: *UR-AI 2022—Proceedings of the 4th Upper-Rhine Artificial Intelligence Symposium: Artificial Intelligence Applications in Medicine and Manufacturing. *Villingen-Schwenningen, Germany, October 19, 2022; 2022:137-145.

[20] Moher D , Liberati A, Tetzlaff J, et al; PRISMA Group. Preferred reporting items for systematic reviews and meta-analyses: the Prisma statement. PLoS Med. 2009;6:e1000097. 19621072 10.1371/journal.pmed.1000097 PMC2707599

[21] Wermter J , Hahn U. An annotated German-language medical text corpus as language resource. In: *LREC 2004—Proceedings of the 4th International Conference on Language Resources and Evaluation*, Lisbon, Portugal, May 24-30, 2004; 2004:473-476.

[22] Müller M , Markó KG, Daumke P, et al Biomedical data mining in clinical routine: expanding the impact of hospital information systems. In: *MedInfo 2007—Proceedings of the 12th World Congress on Health (Medical) Informatics. Building Sustainable Health Systems*, Brisbane, Queensland, Australia, August 20-24, 2007; 2007:340-344 (*Stud Health Technol Inform*, 129). 17911735

[23] Spat S , Cadonna B, Rakovac I, et al Enhanced information retrieval from narrative German-language clinical text documents using automated document classification. In: *eHealth Beyond the Horizon—Get IT There. MIE 2008—Proceedings of the 21st International Congress of the European Federation for Medical Informatics*, Gothenburg, Sweden, 25-28 May 2008; 2008:473-478 (*Stud Health Technol Inform*, 136). 18487776

[24] Kreuzthaler M , Schulz S. Truecasing clinical narratives. In: *User Centred Networked Health Care. MIE 2011—Proceedings of the 23rd Conference of the European Federation of Medical Informatics*, Oslo, Norway, August 28-31, 2011; 2011:589-593 (*Stud Health Technol Inform*, 169). 21893817

[25] Fette G , Ertl M, Wörner A, et al Information extraction from unstructured electronic health records and integration into a data warehouse. In: *INFORMATIK 2012: Was bewegt uns in der/die Zukunft? Proceedings der 42. Jahrestagung der Gesellschaft für Informatik e.V. (GI)*, Braunschweig, Deutschland, September 16-21, 2012; 2012:1237-1251 (*GI-Edition—Lecture Notes in Informatics, P-208*).

[26] Bretschneider C , Oberkampf H, Zillner S, et al Corpus-based translation of ontologies for improved multilingual semantic annotation. In: *SWAIE 2014—Proceedings of 3rd Workshop on Semantic Web and Information Extraction @ COLING 2014*, Dublin, Ireland, August 24, 2014; 2014:1-8.

[27] Bretschneider C , Zillner S, Hammon M. Identifying pathological findings in German radiology reports using a syntacto-semantic parsing approach. In: *BioNLP 2013—Proceedings of the 2013 Workshop on Biomedical Natural Language Processing @ ACL 2013*, Sofia, Bulgaria, August 8, 2013; 2013:27-35.

[28] Toepfer M , Corovic H, Fette G, et al Fine-grained information extraction from German transthoracic echocardiography reports. BMC Med Inform Decis Mak. 2015;15:91. 26563260 10.1186/s12911-015-0215-x PMC4643516

[29] Lohr C , Herms R. A corpus of German clinical reports for ICD and OPS-based language modeling. In: *CLAW 2016—Proceedings of the 6th Workshop on Controlled Language Applications @ LREC 2016*, Portorož, Slovenia, May 28, 2016; 2016:20-23.

[30] Löpprich M , Krauss F, Ganzinger M, et al Automated classification of selected data elements from free-text diagnostic reports in clinical research. Methods Inf Med. 2016;55:373-380. 27406024 10.3414/ME15-02-0019

[31] Roller R , Uszkoreit H, Xu F, et al A fine-grained corpus annotation schema of German nephrology records. In: *ClinicalNLP 2016—Proceedings of the 1st Workshop on Clinical Natural Language Processing @ COLING 2016*, Osaka, Japan, December 11, 2016; 2016:69-77.

[32] Roller R , Seiffe L, Ayach A, et al Information extraction models for German clinical text. In: *ICHI 2020—Proceedings of the [8th] 2020 IEEE International Conference on Healthcare Informatics*, 30 November-3 December 2020 (Virtual Event). 2020:527-528.

[33] Roller R , Seiffe L, Ayach A, et al A medical information extraction workbench to process German clinical text, arXiv: 2207.03885. https://arxiv.org/abs/2207.03885, 2022, preprint: not peer reviewed.

[34] Cotik V , Roller R, Xu F, et al Negation detection in clinical reports written in German. In: *BioTxtM 2016—Proceedings of the 5th Workshop on Building and Evaluating Resources for Biomedical Text Mining @ COLING 2016*, Osaka, Japan, December 12, 2016; 2016:115-124.

[35] Roller R , Rethmeier N, Thomas PE, et al Detecting named entities and relations in German clinical reports. In: *Language Technologies for the Challenges of the Digital Age. GSCL 2017—Proceedings of the 27th International Conference of the German Society for Computational Linguistics and Language Technology*, Berlin, Germany, September 13-14, 2017; 2018:146-154 (*Lecture Notes in Artificial Intelligence*, 10713).

[36] Kreuzthaler M , Oleynik M, Avian A, et al Unsupervised abbreviation detection in clinical narratives. In: *ClinicalNLP 2016—Proceedings of the 1st Workshop on Clinical Natural Language Processing @ COLING 2016*, Osaka, Japan, December 11, 2016; 2016:91-98.

[37] Oleynik M , Kreuzthaler M, Schulz S. Unsupervised abbreviation expansion in clinical narratives. In: *MedInfo 2017—Proceedings of the 16th World Congress on Medical and Health Informatics: Precision Healthcare through Informatics*; Hangzhou, China, August 21-25, 2017; 2017:539-543 (*Stud Health Technol Inform*, 245). 29295153

[38] Krebs J , Corovic H, Dietrich G, et al Semi-automatic terminology generation for information extraction from German chest X-ray reports. In: *German Medical Data Sciences: Visions and Bridges. GMDS 2017—Proceedings of the 62nd Annual Meeting of the German Association of Medical Informatics, Biometry and Epidemiology (gmds e.V.) 2017*, Oldenburg (Oldenburg), Germany, September 17-21, 2017; 2017:80-84 (*Stud Health Technol Inform*, 243). 28883175

[39] Hahn U , Modersohn L, Faller J, et al Final report on the German clinical reference corpus 3000PA. In: *MEDINFO 2023—The Future Is Accessible. Proceedings of the 19th World Congress on Medical and Health Informatics*, Sydney, New South Wales, Australia, July 8-12, 2023. 2024:599-603 (*Stud Health Technol Inform*, 310). 10.3233/SHTI231035 38269879

[40] Hahn U , Matthies F, Lohr C, et al 3000PA: towards a national reference corpus of German clinical language. In: *Building Continents of Knowledge in Oceans of Data—The Future of Co-Created eHealth. MIE 2018—Proceedings of the 29th Conference on Medical Informatics in Europe*, Gothenburg, Sweden, April 24-26, 2018; 2018:26-30 (*Stud Health Technol Inform*, 247). 29677916

[41] Lohr C , Luther S, Matthies F, et al CDA-compliant section annotation of German-language discharge summaries: guideline development, annotation campaign, section classification. In: *AMIA Annu Symp Proc 2018. Data, Technology, and Innovation for Better Health*, San Francisco, California, USA, November 3-7, 2018; 2018:770-779. PMC6371337 30815119

[42] Kolditz T , Lohr C, Hellrich J, et al Annotating German clinical documents for de-identification. In: *MEDINFO 2019—Proceedings of the 17th World Congress on Medical and Health Informatics: Health and Wellbeing e-Networks for All*, Lyon, France, August 25-30, 2019; 2019:203-207 (*Stud Health Technol Inform*, 264). 10.3233/SHTI190212 31437914

[43] Lohr C , Modersohn L, Hellrich J, et al An evolutionary approach to the annotation of discharge summaries. In: *Digital Personalized Health and Medicine. MIE 2020—Proceedings of the 30th Conference on Medical Informatics Europe*, Geneva, Switzerland, April 28-May 1, 2020; 2020:28-32 (*Stud Health Technol Inform*, 270). 10.3233/SHTI200116 32570340

[44] Becker M , Kasper S, Böckmann B, et al Natural language processing of German clinical colorectal cancer notes for guideline-based treatment evaluation. Int J Med Inform. 2019;127:141-146. 31128826 10.1016/j.ijmedinf.2019.04.022

[45] Richter-Pechanski P , Wiesenbach P, Schwab DM, et al A distributable German clinical corpus containing cardiovascular clinical routine doctor’s letters. Sci Data. 2023;10:207. 37059736 10.1038/s41597-023-02128-9 PMC10104831

[46] Richter-Pechanski P , Geis NA, Kiriakou C, et al Automatic extraction of 12 cardiovascular concepts from German discharge letters using pre-trained language models. Digital Health. 2021;7:20552076211057662. 10.1177/20552076211057662 34868618 PMC8637713

[47] Richter-Pechanski P , Amr A, Katus HA, et al Deep learning approaches outperform conventional strategies in de-identification of German medical reports. In: *German Medical Data Sciences: Shaping Change—Creative Solutions for Innovative Medicine. GMDS 2019—Proceedings of the 64th Annual Meeting of the German Association of Medical Informatics*, *Biometry and Epidemiology*, Dortmund, Germany, September 8-11, 2019; 2019:101-109 (*Stud Health Technol Inform*, 267). 10.3233/SHTI190813 31483261

[48] König M , Sander A, Demuth I, et al Knowledge-based best of breed approach for automated detection of clinical events based on German free text digital hospital discharge letters. PLoS One. 2019;14:e0224916. 31774830 10.1371/journal.pone.0224916 PMC6881027

[49] Bressem KK , Papaioannou J-M, Grundmann P, et al MedBert.de: a comprehensive German Bert model for the medical domain. Expert Systems with Applications. 2024;237:121598.

[50] Bressem KK , Adams LC, Gaudin RA, et al Highly accurate classification of chest radiographic reports using a deep learning natural language model pre-trained on 3.8 million text reports. Bioinformatics. 2020;36:5255-5261. 10.1093/bioinformatics/btaa668 32702106

[51] Grundel B , Bernardeau M-A, Langner H, et al Merkmalsextraktion aus klinischen Routinedaten mittels Text-Mining. Der Ophthalmologe. 2021;118:264-272. 32725541 10.1007/s00347-020-01177-4

[52] Irschara K. Using a corpus-assisted discourse studies approach to analyse gender: a case study of German radiology reports. Gender a Výzkum. 2022;23:114-139.

[53] Irschara K , Posch C, Waldner B, et al Building the MedCorpInn corpus: issues and goals In: Posch C, Irschara K, Rampl G, eds. Wort—Satz—Korpus: Multimethodische digitale Forschung in der Linguistik. Innsbruck University Press; 2022:163-191.

[54] Madan S , Zimmer FJ, Balabin H, et al Deep learning-based detection of psychiatric attributes from German mental health records. Int J Med Inform. 2022;161:104724. 35279550 10.1016/j.ijmedinf.2022.104724

[55] Trienes J , Schlötterer J, Schildhaus H-U, et al Patient-friendly clinical notes: towards a new text simplification dataset. In: *TSAR 2022—Proceedings of the [1st] Workshop on Text Simplification, Accessibility, and Readability @ EMNLP-2022*. December 8, 2022 (Virtual Event); 2022:19-27.

[56] Llorca I , Borchert F, Schapranow M-P. A meta-dataset of German medical corpora: harmonization of annotations and cross-corpus NER evaluation. In: *ClinicalNLP 2023—Proceedings of the 5th Workshop on Clinical Natural Language Processing @ ACL 2023*, Toronto, Ontario, Canada, July 14, 2023; 2023:171-181.

[57] Meineke F , Modersohn L, Loeffler M, et al Announcement of the German Medical Text Corpus Project (GeMTeX). In: *Caring is Sharing—Exploiting the Value in Data for Health and Innovation. Proceedings of [the 33rd Medical Informatics Europe Conference] MIE 2023*, Gothenburg, Sweden, May 22-25, 2023; 2023:835-836 (*Stud Health Technol Inform*, 302). 10.3233/SHTI230283 37203512

[58] Idrissi-Yaghir A , Dada A, Schäfer H, et al Comprehensive study on German language models for clinical and biomedical text understanding. In: *LREC-COLING 2024—Proceedings of the 2024 Joint International Conference on Computational Linguistics, Language Resources and Evaluation*, Torino, Italia, May 20-25, 2024; 2024:3654-3665.

[59] Baumgartner M , Kreiner K, Wiesmüller F, et al Masketeer: an ensemble-based pseudonymization tool with entity recognition for German unstructured medical free text. Future Internet. 2024;16:281.

[60] Plagwitz L , Neuhaus P, Yildirim K, et al Zero-shot LLMs for named entity recognition: targeting cardiac function indicators in German clinical texts. In: *German Medical Data Sciences 2024*. *Health—Thinking, Researching and Acting Together. Proceedings of the 69th Annual Meeting of the German Association of Medical Informatics, Biometry, and Epidemiology e.V*. *(gmds) 2024*, Dresden, Germany, September 8-13, 2024; 2024:228-234 (*Stud Health Technol Inform*, 317). 10.3233/SHTI240861 39234726

[61] Becker M , Böckmann B. Extraction of UMLS^®^ concepts using Apache cTakes™ for German language. In: *Health Informatics Meets eHealth. Predictive Modeling in Healthcare—From Prediction to Prevention. Proceedings of the 10th eHealth2016 Conference*, Vienna, Austria, May 24-25, 2016; 2016:71-76 (*Stud Health Technol Inform*, 223). 27139387

[62] Frei J , Frei-Stuber L, Kramer F. GerNERMed++: semantic annotation in German medical NLP through transfer-learning, translation and word alignment. J Biomed Inform. 2023;147:104513. 37838290 10.1016/j.jbi.2023.104513

[63] Frei J , Kramer F. GerNERMed: an open German medical NER model. Software Impacts. 2022;11:100212.

[64] Frei J , Kramer F. German medical named entity recognition model and data set creation using machine translation and word alignment: algorithm development and validation. JMIR Form Res 2023;7:e39077. 36853741 10.2196/39077 PMC10015355

[65] Lohr C , Buechel S, Hahn U. Sharing copies of synthetic clinical corpora without physical distribution: a case study to get around IPRs and privacy constraints featuring the German JSynCC corpus. In: *LREC 2018—Proceedings of the 11th International Conference on Language Resources and Evaluation*, Miyazaki, Japan, May 7-12, 2018; 2018:1259-1266.

[66] Modersohn L , Schulz S, Lohr C, et al GraSCCo: the first publicly shareable, multiply-alienated German clinical text corpus. In: *German Medical Data Sciences 2022—Future Medicine: More Precise, More Integrative, More Sustainable! Proceedings of the Joint Conference of the 67th Annual Meeting of the GMDS & 14th Annual Meeting of the TMF*, 21-25 August 2022 (Virtual Event); 2022:66-72 (*Stud Health Technol Inform*, 296). 10.3233/SHTI220805 36073490

[67] Frei J , Kramer F. Annotated dataset creation through large language models for non-English medical NLP. J Biomed Inform. 2023;145:104478. 37625508 10.1016/j.jbi.2023.104478

[68] Brown RD. Corpus-driven splitting of compound words. In: *Proceedings of the 9th Conference on Theoretical and Methodological Issues in Machine Translation of Natural Languages: Papers*, Keihanna, Japan, March 13-17, 2002; 2002:3.

[69] Volk M , Ripplinger B, Vintar Š, et al Semantic annotation for concept-based cross-language medical information retrieval. Int J Med Inform. 2002;67:79-112. 10.1016/s1386-5056(02)00058-8 12460635

[70] Markó KG , Daumke P, Schulz S, et al Cross-language MeSH indexing using morpho-semantic normalization. In: *AMIA Annu Symp Proc 2003. Biomedical and Health Informatics: From Foundations to Applications*, Washington, DC, USA, November 8-12, 2003; 2003:425-429. PMC1480043 14728208

[71] Rogati M , Yang Y. Customizing parallel corpora at the document level. In: *ACL '04—Proceedings of the 42nd Annual Meeting of the Association for Computational Linguistics: Interactive Poster and Demonstration Sessions*, Barcelona, Spain, July 21-26, 2004; 2004:110-113.

[72] Morin É , Daille B. Revising the compositional method for terminology acquisition from comparable corpora. In: *COLING 2012—Proceedings of the 24th International Conference on Computational Linguistics*, Mumbai, India, December 8-15, 2012; 2012:1797-1810.

[73] Hellrich J , Clematide S, Hahn U, et al Collaboratively annotating multilingual parallel corpora in the biomedical domain: some Mantras. In: *LREC 2014—Proceedings of the 9th International Conference on Language Resources and Evaluation*, Reykjavik, Iceland, May 26-31, 2014. 2014:4033-4040.

[74] Kors JA , Clematide S, Akhondi SA, et al A multilingual gold-standard corpus for biomedical concept recognition: the Mantra GSC. J Am Med Inform Assoc. 2015;22:948-956. 25948699 10.1093/jamia/ocv037 PMC4986661

[75] Bojar O , Haddow B, Mareček D, et al *HimL D1.1: Report on Building Translation Systems for Public Health Domain*. Version 1.0. 2017. European Union’s Horizon 2020 Research and Innovation Programme, grant No 644402. Accessed April 1, 2025. https://www.himl.eu/files/himl/D1.1-report-on-building-translation-systems.pdf

[76] Mouriño García MA , Pérez Rodríguez R, Rifón LA. Leveraging Wikipedia knowledge to classify multilingual biomedical documents. Artif Intell Med. 2018;88:37-57. 29730047 10.1016/j.artmed.2018.04.007

[77] Villena F , Eisenmann U, Knaup P, et al On the construction of multilingual corpora for clinical text mining. In: *Digital Personalized Health and Medicine. MIE 2020—Proceedings of the 30th Conference on Medical Informatics Europe*, Geneva, Switzerland, April 28-May 1, 2020; 2020:347-351 (*Stud Health Technol Inform*, 270). 10.3233/SHTI200180 32570404

[78] Borchert F , Lohr C, Modersohn L, et al GGPOnc 2.0—the German Clinical Guideline Corpus for Oncology: curation workflow, annotation policy, baseline NER taggers. In*: LREC 2022—Proceedings of the 13th International Conference on Language Resources and Evaluation*, Marseille, France, June 20-25, 2022; 2022:3650‑3660.

[79] Borchert F , Lohr C, Modersohn L, et al GGPOnc: a corpus of German medical text with rich metadata based on clinical practice guidelines. In: *LOUHI 2020—Proceedings of the 11th International Workshop on Health Text Mining and Information Analysis @ EMNLP 2020*. November 20, 2020 (Virtual Event); 2020:38-48.

[80] Lentzen M , Madan S, Lage-Rupprecht V, et al Critical assessment of transformer-based AI models for German clinical notes. JAMIA Open. 2022;5:ooac087. 36380848 10.1093/jamiaopen/ooac087 PMC9663939

[81] Arnold S , Schneider R, Cudré-Mauroux P, et al Sector: a neural model for coherent topic segmentation and classification. Trans Assoc Comput Linguist. 2019;7:169-184.

[82] Seiffe L , Marten O, Mikhailov M, et al From witch’s shot to music making bones: resources for medical laymen to technical language and vice versa. In: *LREC 2020—Proceedings of the 12th International Conference on Language Resources and Evaluation,* Marseille, France, May 11-16, 2020; 2020:6185-6192.

[83] Wolfer S , Koplenig A, Michaelis F, et al Tracking and analyzing recent developments in German-language online press in the face of the coronavirus crisis: cOWIDplus analysis and cOWIDplus viewer. Int J Corpus Linguist. 2020;25:347-359.

[84] Beck T , Lee J-U, Viehmann C, et al Investigating label suggestions for opinion mining in German Covid-19 social media. In: *ACL-IJCNLP 2021—Proceedings of the 59th Annual Meeting of the Association for Computational Linguistics & 11th International Joint Conference on Natural Language Processing*. Volume 1: Long Papers. August 1-6, 2021 (Virtual Event); 2021:1-13.

[85] Mattern J , Qiao Y, Kerz, et al Fang-Covid: a new large-scale benchmark dataset for fake news detection in German. In: *FEVER 2021—Proceedings of the 4th Workshop on Fact Extraction and VERification @ EMNLP 2021*. November 10, 2021 (Virtual Event); 2021:78-91.

[86] Raithel L , Thomas PE, Roller R, et al Cross-lingual approaches for the detection of adverse drug reactions in German from a patient’s perspective. In: *LREC 2022—Proceedings of the 13th International Conference on Language Resources and Evaluation*, Marseille, France, June 20-25, 2022; 2022:3637-3649.

[87] Raithel L , Yeh H-S, Yada S, et al A dataset for pharmacovigilance in German, French, and Japanese: annotating adverse drug reactions across languages. In: *LREC-COLING 2024—Proceedings of the 2024 Joint International Conference on Computational Linguistics, Language Resources and Evaluation*, Torino, Italia, May 20-25, 2024; 2024:395-414.

[88] Heinrich P , Blombach A, Dang BMD, et al Automatic identification of COVID-19-related narratives in German Telegram channels and chats. In: *LREC-COLING 2024—Proceedings of the 2024 Joint International Conference on Computational Linguistics, Language Resources and Evaluation*, Torino, Italia, May 20-25, 2024; 2024:1932-1943.

[89] Vladika J , Schneider P, Matthes F. HealthFC: verifying health claims with evidence-based medical fact-checking. In: *LREC-COLING 2024—Proceedings of the 2024 Joint International Conference on Computational Linguistics, Language Resources and Evaluation*; Torino, Italia, May 20-25, 2024; 2024:8095-8107.

[90] Pedrini G. Between Plain Language and Einfache Sprache: A Corpus Analysis of Layperson Summaries of Clinical Trials in English, German, and Italian. Frank & Timme Verlag; 2024.

[91] Frei J , Kramer F. Creating ontology-annotated corpora from Wikipedia for medical named-entity recognition. In: *BioNLP 2024—Proceedings of the 23rd Meeting of the ACL Special Interest Group on Biomedical Natural Language Processing: Workshop and Shared Tasks @ ACL 2024*, Bangkok, Thailand, August 16, 2024; 2024:570-579.

[92] Faessler E , Hellrich J, Hahn U. Disclose models, hide the data: how to make use of confidential corpora without seeing sensitive raw data. In: *LREC 2014—Proceedings of the 9th International Conference on Language Resources and Evaluation*, Reykjavik, Iceland, May 26-31, 2014; 2014:4230-4237.

[93] Hellrich J , Matthies F, Faessler E, et al Sharing models and tools for processing German clinical texts. In: *Digital Healthcare Empowering Europeans. MIE 2015—Proceedings of the 26th Conference on Medical Informatics in Europe*, Madrid, Spain, May 27-29, 2015; 2015:734-738 (*Stud Health Technol Inform*, 210). 25991250

[94] Fries JA , Weber L, Seelam N, et al BigBIO: a framework for data-centric biomedical natural language processing. In: *Advances in Neural Information Processing Systems 35—NeurIPS 2022. Proceedings of the 36th Annual Conference on Neural Information Processing Systems*, New Orleans, Louisiana, USA, November 28-December 9, 2022; 2022:25792-25806.

[95] Suominen H , Salanterä S, Velupillai S, et al Overview of the ShARe/CLEF eHealth Evaluation Lab 2013. In: Forner P, Müller H, Parades R, et al., eds. *Information Access Evaluation. Multilinguality, Multimodality, and Visualization. CLEF 2013—Proceedings of the 4th International Conference of the CLEF Initiative*. Valencia, Spain, September 23-26, 2013. Springer; 2013:212-231 (*Lecture Notes in Computer Science*, 8138).

[96] Henry S , Buchan K, Filannino M, et al 2018 n2c2 shared task on adverse drug events and medication extraction in electronic health records. J Am Med Inform Assoc. 2020;27:3-12. 31584655 10.1093/jamia/ocz166 PMC7489085

[97] Yang J , Jin H, Tang R, et al Harnessing the power of LLMs in practice: a survey on ChatGPT and beyond. ACM Transactions on Knowledge Discovery from Data. 2024;18:160.

[98] Zhou C , Li Q, Li C, et al A comprehensive survey on pretrained foundation models: a history from Bert to ChatGPT. Int J Mach Learn & Cyber. 2024. Accessed April 1, 2025. 10.1007/s13042-024-02443-6

[99] Şerbetçi O , Leser U. Applicability of models trained on generated clinical German datasets on out-domain data. In: *LWDA 2023—Proceedings of the Conference on* “*Lernen*, *Wissen, Daten, Analysen*,” Marburg, Germany, October 9-11, 2023; 2023:521-525.

[100] Kweon S, Kim J, Kim J, et al Publicly shareable clinical large language model built on synthetic clinical notes. In: *Findings of the Association for Computational Linguistics – ACL 2024*, Bangkok, Thailand, August 11-16, 2024. (Hybrid Event). 2024:5148-5168.

[101] Hiebel N, Ferret O, Fort K, et al Can synthetic text help clinical named entity recognition? A study of electronic health records in French. In: *EACL 2023 –Proceedings of the 17th Conference of the European Chapter of the Association for Computational Linguistics*, Dubrovnik, Croatia, May 2-6, 2023 (Hybrid Event). 2023:2320-2338.

[102] Brekke PH, Rama T, Pilán I, et al Synthetic data for annotation and extraction of family history information from clinical text. J Biomed Semant. 2021;12:11. 10.1186/s13326-021-00244-2 PMC8278746 34261535

[103] Li J, Zhou Y, Jiang X, et al Are synthetic clinical notes useful for real natural language processing tasks: a case study on clinical entity recognition. J Am Med Inform Assoc. 2021;28:2193-2201. 34272955 10.1093/jamia/ocab112 PMC8449609

[104] Ive J, Viani N, Kam J, et al Generation and evaluation of artificial mental health records for natural language processing. npj Digit Med 2020;3:69. 32435697 10.1038/s41746-020-0267-x PMC7224173

[105] Pan X , Zhang M, Ji S, et al Privacy risks of general-purpose language models. In: *SP 2020—Proceedings of the 2020 IEEE Symposium on Security and Privacy*, San Francisco, California, USA, May 18-21, 2020; 2020:1314-1331.

[106] Carlini N , Tramèr F, Wallace E, et al Extracting training data from large language models. In: *USENIX Security '21—Proceedings of the 30th USENIX Security Symposium*. August 11–13, 2021 (Virtual Event); 2021:2633-2650.

[107] Larbi IBC , Burchardt A, Roller R. Clinical text anonymization, its influence on downstream NLP tasks and the risk of re-identification. In: *Proceedings of the Student Research Workshop @ EACL 2023*, Dubrovnik, Croatia, May 2-4, 2023; 2023:105-111.

[108] Ayers JW , Caputi TL, Nebeker C, et al Don’t quote me: reverse identification of research participants in social media studies. npj Digital Medicine. 2018;1:30. 31304312 10.1038/s41746-018-0036-2 PMC6550214

[109] Chhikara P , Pasupulety U, Marshall J, et al Privacy aware question-answering system for online mental health risk assessment. In: *BioNLP 2023—Proceedings of the 22nd Workshop on Biomedical Language Processing (BioNLP) & BioNLP Shared Tasks (BioNLP-ST) @ ACL 2023*, Toronto, Ontario, Canada, 13 July 2023; 2023:215-222.

[110] Adams LC , Truhn D, Busch F, et al (2023). Leveraging GPT-4 for post hoc transformation of free-text radiology reports into structured reporting: a multilingual feasibility study. Radiology, 2023;307:e230725. 10.1148/radiol.230725 37014240

[111] Richter-Pechanski P , Wiesenbach P, Schwab DM, et al Few-shot and prompt training for text classification in German doctor’s letters. In: *Caring is Sharing—Exploiting the Value in Data for Health and Innovation*. *Proceedings of [the 33rd Medical Informatics Europe Conference] MIE 2023*, Gothenburg, Sweden, 22-25 May 2023; 2023:819-820 (*Stud Health Technol Inform*, 302). 10.3233/SHTI230275 37203504

[112] Dada A , Chen A, Peng C, et al On the impact of cross-domain data on German language models. In: *Findings of the Association for Computational Linguistics—EMNLP 2023*, Singapore, Singapore, December 6-10, 2023; 2023:13801-13813.

[113] Heilmeyer F , Böhringer D, Reinhard T, et al Viability of open large language models for clinical documentation in German health care: real-world model evaluation study. JMIR Med Inform 2024;12:e59617. 39195570 10.2196/59617 PMC11373371

[114] Lohr C , Hahn U. DoPA Meter: a tool suite for metrical document profiling and aggregation. In: *EMNLP 2023—Proceedings of the 2023 Conference on Empirical Methods in Natural Language Processing: System Demonstrations*, Singapore, Singapore, December 6-10, 2023; 2023:218-228.

[115] Kara E , Zeen T, Gabryszak A, et al A domain-adapted dependency parser for German clinical text. In: *KONVENS 2018—Proceedings of the 14th Conference on Natural Language Processing*. Vienna, Austria, September 19-21, 2018. Vienna: Austrian Academy of Sciences (ÖAW); 2018:12-17.

[116] McMillan-Major A , Osei S, Rodriguez JD, et al Reusable templates and guides for documenting datasets and models for natural language processing and generation: a case study of the HuggingFace and GEM data and model cards. In: *GEM 2021—Proceedings of the 1st Workshop on Natural Language Generation, Evaluation, and Metrics @ ACL 2021*, August 5-6, 2021 (Virtual Event); 2021:121-135.

[117] Gebru T , Morgenstern J, Vecchione B, et al Datasheets for datasets. Commun ACM. 2021;64:86-92.

