# Supplementary material for: Clinical document corpora—real ones, translated and synthetic substitutes, and assorted domain proxies: a survey of diversity in corpus design, with focus on German text data
Source: JAMIA Open. 2025 May 14;8(3):ooaf024. doi: 10.1093/jamiaopen/ooaf024 (PMC12077144; doi:10.1093/jamiaopen/ooaf024)
Supplement: ooaf024_Supplementary_Data [file ooaf024_supplementary_data.zip › Supplement_Tables_23456_OUP_ooaf024_V2.docx]

**Supplementary Material**

1. **Tables of German-Language Clinical/Medical Corpora**

The five tables contained in this section are grouped, in decreasing order, by typological homogeneity and text genre similarity relative to German-language clinical reports and notes:

- **Table 2** features *real* *clinical* corpora composed of authentic textual material (e.g., original discharge summaries, pathology or radiology reports, etc.),
- **Table 3** lists clinical corpora that have been *translated* from a foreign language (typically, American English) to German,
- **Table 4** introduces *synthetic* clinical corpora with fictitious descriptions of virtual patients, yet in the format of real clinical documents,
- **Table 5** assembles non-clinical corpora with medical contents though, collected from *scholarly publications* hosted in digital libraries (typically, PubMed), publishers’ web sites, or even science-focused newspaper articles,
- **Table 6** contains non-clinical corpora that were built from *social media* data (tweets, chats, blogs, etc.) or *encyclopedic articles* (typically, Wikipedia), all dealing with medical topics.

Each of these tables is structured following a common column format:

- The first column contains the *name* of the corpus (if explicitly introduced in the cited publications) or a pseudo name (first author plus publication year), the *citation*, and the *year of publication* (the rows are ordered in ascending order by year),
- The second column specifies the *number of documents* in the corpus,
- The third column indicates the *number of tokens* in the corpus,
- The fourth column lists the *document type(s) or text genre(s)* incorporated in the corpus, including the specific medical domain the documents deal with,
- The fifth column provides detailed information of the *metadata* that was added to the documents, i.e., the annotation types and number of associated annotation items provided; in addition, we indicate whether
  - *entity normalization*, i.e., grounding of the entity instances in some (medical) terminology or ontology, was carried out (and, if so, mention the chosen concept system),
  - *annotation guidelines* that were used for the manual generation of metadata are accessible (e.g., in the supplementary material section of the article, or in an open access data portal, such as GitHub),
  - *inter-annotator agreement (IAA)* was measured by some canonical metric and the resulting scores are reported as individual values per type or in an aggregated macro form over all types,
- The sixth column marks the availability of the corpus, i.e., whether it is *inaccessible* (classified): ⚫, *publicly available via contract-based access*, typically based on a Data Use Agreement (DUA), or other types of private commitments or institutional negotiations: **🖂** ), or *publicly available without any restrictions*: **✓**; as a special option, ◆ marks the availability of language models derived from a specific corpus.^[[1]](#footnote-1)^

“noi” indicates that “no information” about specific quantitative data are reported in the publication; “n/a” indicates that a specific categorical information is “not applicable” (e.g., IAA data for automatically generated (silver standard) metadata or data extracted from the structured portion of the EHR segment of the clinical information system).

Descriptions that are relevant for a particular category (say, real clinical reports) are colored in black whereas grey colored areas apply to other categories of the same corpus (e.g., if the corpus contains Wikipedia data, as well, which are discussed in the table relating to distant domain proxies). Accordingly, the reader always gets a complete picture of the textual variety of the corpus without losing focus.

In **Appendix A** we propose a more elaborate corpus datasheet, the template for a *corpus card*, with mandatory and (desirable) optional description categories for clinical/medical corpora (see Table A1).

| **Corpus /**  **Citation – Year** | **Docu-ments** | **Tokens**  (in 1,000 =1k) | **Clinical**  **Document Types**  **(Text Genres)** | **Metadata** | **Avail-ability**  ⚫ **Corpus**  ◆ **Model** |
| --- | --- | --- | --- | --- | --- |
| **FraMed**  [21] – 2004 | noi  (~6,500 sentences) | 100k | Various clinical report types (discharge, pathology, histol-ogy, and surgery reports),  a medical textbook, and  Web documents taken from a consumer health care portal (netdoktor) | **Annotation Types**  Sentence & token splits, parts of speech (PoS)  **Entity Normalization:** Y (medically adapted STTS for PoS annotation)  **Annotation Guideline:** N  **IAA Measurement:**  Y | ⚫  ◆  FraMed model as part of JCoRe^[[2]](#footnote-2)^  [92,93] |
| **Müller-07**  [22] – 2007 | ~ 30,000 | noi | Mainly discharge letters, but also surgical reports, immunodermatological  findings and other narrative  reports of clinical results  (dermatology) | none | ⚫ |
| **Spat-08**  [23] – 2008 | 1,500  subset from  18k | noi | 26 clinical document types from 8 medical fields (vascular & casualty surgery, internal medicine, neurology, anaesthesia, intensive care, radiology, physiotherapy) | **Annotation Types**  Classification into document types and medical fields  **Entity Normalization:** N  **Annotation Guideline:** N  **IAA Measurement:**  N | ⚫ |
| **Kreuz-thaler-11**  [24] – 2011 | 3,542 | 84k | Pathology reports | **Annotation**  (Automatic) rewriting of fully capitalized texts as mixed capitalized and lower-cased texts (following German orthography rules)  **Entity Normalization:** n/a  **Annotation Guideline:** n/a  **IAA Measurement:**  n/a | ⚫ |
| **Fette-12**  [25] – 2012 | 544  subset from  193k | noi | Clinical reports from 5 clinical domains (echocardiography, ECG, lung function, X-ray thorax, bicycle stress test) | **Annotation Types**  Automatic extraction of attribute-value pairs from the 5 clinical domains  **Entity Normalization:** Y (local terminology)  **Annotation Guideline:** N  **IAA Measurement:**  N | ⚫ |
| **Bret-schnei-der-13**  [27] – 2013 | 174  subset from  2,7k | 28k | Radiology reports  (lymphoma) | **Annotation**  classification into “*pathological*“ or “*non-pathological*“ sentences  **Entity Normalization:** N  **Annotation Guideline:** N  **IAA Measurement:**  N | ⚫ |
| **Bret-schnei-der-14**  [26] – 2014 | 2,713 | 347k | Radiology reports (lymphoma) | **Annotation Types**  **(Annotated items)**  Automatic concept annotation with (ma-chine-translated) German RadLex terms  (Σ: 148k tokens (= 42.6 %))  **Entity Normalization:** Y (RadLex)  **Annotation Guideline:** n/a  **IAA Measurement:**  n/a | ⚫ |
| **Toepfer-15**  [28] – 2015 | 140  subset from  69k/70k | noi | (Transthoracic) echocardio-graphy reports | **Annotation Types**  **(Annotated items)**  Automatic extraction of 440 attribute-value pairs from the echocardiography domain (e.g., attributes: *Aortic Valve, Mitral Valve, Tricuspid Valve, regurgitation (aortic), Aorta, Stenosis, Diastolic Function, Left/Right Ventricle*; values: *present, absent, severe*)  (Σ: 6,2k)  **Entity Normalization:** Y (local terminology mapped to a guideline for German trans-thoracic echocardiography reports)  **Annotation Guideline:** N  **IAA Measurement:**  Y | ⚫ |
| **Lohr-16**  [29] – 2016 | 450  subset from  22,4k  5,8m | 266k  125,9m | Operative reports  (digestive tract)  (Fragments of) newspaper articles with medical content extracted from DWDS (*Digitales Wörterbuch der Deutschen Sprache*) | **Annotation Types**  *Diagnoses*, *Procedures*  **Entity Normalization:** Y (ICD for diagnoses, OPS for executed procedures)  **Annotation Guideline:** n/a (extracted from EPR as gold standard)  **IAA Measurement:**  n/a  Mentions of 400 medical terms, such as *“patient”, “surgery”, “ambulance”*, etc.  **Entity Normalization:** N  **Annotation Guideline:** n/a  **IAA Measurement:**  n/a | ⚫ |
| **Löp-prich-16**  [30] – 2016 | 737  (para-graphs only) | noi | Main diagnosis paragraphs split from discharge sum-maries (*oncology*: *multiple myeloma*) | **Annotation Types**  **(Annotated items)**  *Diagnosis* (0,9k), *State of Disease* (specific data elements characteristic for multiple myeloma; 7,7k)  (Σ: 8,6k)  **Entity Normalization:** N  **Annotation Guideline:** N  **IAA Measurement:**  Y | (**✓** \| )  ⚫^[[3]](#footnote-3)^ |
| **Roller-16**  [31] – 2016 | 118  + 1,607  = 1,725 | 90k  + 68k  = 158k | Discharge summaries &  clinical notes (nephrology) | **Annotation Types**  **(Annotated items)**  23 entity types, grouped into 7 major categories:  [Time: *Date, Temporal Course*;  Person/Body: *Person, Body Part, Tissue, Body Fluid, Localization*;  Process: *Process*;  Condition: *State of Health, Medical Condition, Diag-nostic/Lab Procedure, Medical Specification, Degree, Type*;  Therapy: *Medical Device, Medication, Biological Chem-istry, Treatment*, *Measurement*;  Structure: *Structure Element*;  Factuality: *Modality Positive, Modality Negation, Modality Vagueness*]  **Entity Normalization:** Y (UMLS)  **Annotation Guideline:** N (scheme only)  **IAA Measurement:**  N | ⚫ |
| **Kreuz-taler-16**  [36] – 2016 | 1,696 | noi | Discharge letters (dermatology) | **Annotation**  **(Annotated items)**  abbreviated word forms  (Σ: 2,3 k)  **Entity Normalization:** N  **Annotation Guideline:** N  **IAA Measurement:**  Y | ⚫ |
| **Cotik-16**  [34] – 2016 | 8  + 175  = 183 | 6,2k  + 6,7k  = 12,9k | Discharge summaries &  clinical notes (nephrology) | **Annotation Types**  **(Annotated items)**  *Negation* (0,4k) & *Factuality*: *affirmed* (0,6k), *speculated* (<0,1k) of *Findings*  (Σ: 1,1k)  **Entity Normalization:** Y (UMLS)  **Annotation Guideline:** N (schema only)  **IAA Measurement:**  N | ⚫ |
| **Seuss-17**  [16] – 2017 | 1,400  [subset from  4,671  + 2,804  + 1,008  + 6,223  = 14,706 | ~5,000k  ~50,000k | pathology reports  medical reports (Gynecology)  operative reports (Gynecology)  radiology reports | **Annotation Types**  **(Annotated items)**  9 Personally Identifiable Information (PII) types  [*Name, Age, Contact, Address, Date of birth/surgery/ examination, Medical ID,* etc.]  (Σ: 23,5k)  **Entity Normalization:** N  **Annotation Guideline:** N  **IAA Measurement:**  N | ⚫ |
| **Oleynik-17**  [37] – 2017 | 30,000 | noi | discharge summaries (cardiology) | none  (200 abbreviations) | ⚫ |
| **Roller-18**  [35] – 2018 | 626  (subset from  [31]) | 26,5k*  (*estimated from averages) | Clinical notes &  discharge summaries  (nephrology) | **Annotation Types**  **(Annotated items)**  8 named entity types:  [*Medical Condition:* *Symptom*, *Finding*, *Diagnosis* (2,5k), *Treatment* (1,7k), *State of Health* (1,5k), *Medi-cation* (1,2k), *Biological Process* (1,2k), *Body Part / Or-gan* (0,8k), *Medical Specification* (0,8k), Locality (1,9k)]  (Σ: 9,7k)  5 relation types:  [*hasState* (0,4k), *Involves* (0,4k), *hasMeasure* (0,4k), *isLocated* (0,2k), *isSpecified* (0,1k)]:  (Σ: 1,5k)  **Entity Normalization:** N  **Annotation Guideline:** N (scheme only)  **IAA Measurement:**  N | ⚫ |
| **Krebs-17**  [38] – 2017 | 100  subset from  3,000 | noi | Radiology reports  (chest) | 1. Semi-automatic acquisition of a local clinical terminology composed of 258 attributes for processing radiology reports; 2. Value categories for attributes: *negation*, *laterality* (right, left, both sides), *location*, *degree of severity*, *condition-after*, & *progression note*. 3. Automatic extraction of 735 attribute-value pairs.   **Entity Normalization:** Y (local terminology)  **Annotation Guideline:** n/a  **IAA Measurement:**  n/a | ⚫ |
| **3000PA 1.0**  [40] – 2018 | 2,360  (from 3 different clinical sites) | 3,997k | (mostly) Discharge sum-maries, few transfer letters | **Annotation Types**  1 *Medication* entity + 5 *Medication* relation types  [*Medication*/*Drug: Dosage, Mode, Frequency, Duration, Medical Reason*]  **Entity Normalization:** N  **Annotation Guideline:** N (scheme only)  **IAA Measurement:**  Y | ⚫ |
| **3000PA 2.0 (1000PA-J)**  [41] – 2018 | 1,106  subset from  3000PA | 1,500k | (mostly) Discharge sum-maries, few transfer letters | **Annotation Types**  **(Annotated items)**  18 *Section Heading* types  [*Salutation* (12,9k), *Anamnesis* (0,6k): *Patient history (6,0k)* & *Family history* (<0,1k), *Diagnosis* (4,0k): *Admission diagnosis* (9,2k) & *Discharge diagnosis* (4,8k), *Hospital discharge studies summary* (87,1k), *Procedures* (3,9k), *Allergies intolerances risks* (0,2k), *Medication* (0,4k): *Admission medication* (0,1k) & *Medication during stay* (0,5k) & *Discharge medication* 11,6k), *Hospital course* (19,8k), *Plan of care* (3,6k), *Final remarks* (4,8k), *Supplements* (1,0k)]  (Σ: 171k)  **Entity Normalization:** Y (CDA-compliant)  **Annotation Guideline:** N (scheme only)  **IAA Measurement:**  Y | ⚫ |
| **Becker-19**  [44] – 2019 | 820  + 817  + 107  + 326  + 20  + 423  = 2,513  subset from  5,506 | noi | (Mixed) clinical reports:  medical reports,  radiology reports,  microbiology reports,  pathology reports,  virology reports, and  tumor board protocols | **Annotation Types**  **(Annotated items)**  11 named entity types, attributes and values related to *colorectal cancer*  [*ICD-Code* (0,4k), *TNM staging (0,6k), distance measurements (0,1k), microsatellite instability (0,1k), resection potential (0,3k), mutation status* (0,2k), *intensive therapy* (< 0,1k), *large tumor burden* (< 0,1k)*, rapid progress* (< 0,1k)*, tumor symptoms* (< 0,1k)*, organ complications* (0,2k]  (Σ: 2,0k)  **Entity Normalization:** Y (UMLS)  **Annotation Guideline:** N (scheme only)  **IAA Measurement:**  Y | ⚫ |
| **3000PA 3.0**  **(1000PA-J)**  [42] – 2019 | 1,106  subset from  3000PA | 1,400k | (mostly) Discharge sum-maries, few transfer letters | **Annotation Types**  **(Annotated items)**  13 *PII* types  [*Age* (0,5k), *Contact (phone, email, URL;* 0,6k*), Date* (20,6k), *Birthdate (1,1k), ID (patient, e.g., EPR number;* 0,4k*; Typist*; 0,7k*), Location (physical address*; 5,4k*), Medical Unit (hospital or department name*; 6,2k*), Person* (<0,1k), *Patient* (3,2k), *Relative* (<0,1k)*, Staff* (5,2k), *Other* (0,2k)]  (Σ: 44,2k)  **Entity Normalization:** N  **Annotation Guideline:** N (scheme only)  **IAA Measurement:**  Y | ⚫ |
| **Richter-Pechan-ski-19**  [47] – 2019 | 113 | 107k | Medical reports  (cardiology) | **Annotation Types**  **(Annotated items)**  8 *PII* types  [*person, location, date, phone, organization, title, salutation, zip code*]:  (Σ: 5,2k)  **Entity Normalization:** N  **Annotation Guideline:** N  **IAA Measurement:**  N | ⚫ |
| **König-19**  [48] – 2019 | 1,982 | 2,001k | Discharge summaries  (osteoporosis) | **Annotation Types**  **(Annotated items)**  1 *Drug-Disease* relation  [“*proton-pump inhibitor use – osteoporosis*”] (2,0k), including concept recognition for *PPI* and *osteoporosis*  [extracted from the hospital-internal study database as gold standard]  **Entity Normalization:** Y (Wingert Nomenclature)  **Annotation Guideline:** n/a  **IAA Measurement:**  n/a | ⚫ |
| **3000PA 4.0**  **(1000PA-J)**  [43] – 2020 | 1,106  subset from  3000PA | 1,500k | (mostly) Discharge sum-maries, few transfer letters | **Annotation Types**  **(Annotated items)**  3 named entity (NE) types  [*Diagnosis* (55k), *Findings* (155k), *Symptoms* (8k)] &  3 attributes of NE types  [*Time* (previous, recurrent, uncertain), *Modality* (suspected, excluded, uncertain), *Complexity*]  **Entity Normalization:** N  **Annotation Guideline:** N  **IAA Measurement:**  Y | ⚫ |
| **Bressem-20**  [50] – 2020 | 5,783  subset from  3,8m  radiology reports used for model pre-training | 399k*  (*estimated from averages)  416m | Radiology reports  (chest radiographs, chest CT scans) | **Annotation Types**  **(Annotated items)**  9 *Finding* types, incl. *Medical Devices*  [*Congestion* (1,5k), *Opacity (e.g., pneumonia, dystelec-tasis*; 3,1k*), Effusion* (2,5k), *Pneumothorax* (0,4k);  *Central Venous Catheters* (3,0k), *Gastric Tube* (1,3k), *Thoracic Drain* (1,1k), *Tracheal Tube* (2,1k), *Misplaced Medical Device* (0,2k)]  (Σ: 15k)  **Entity Normalization:** N  **Annotation Guideline:** Y (see Supplement)  **IAA Measurement:**  Y | ⚫  ◆  RAD-BERT model^[[4]](#footnote-4)^ |
| **Roller-20**  [32] – 2020 | 118  + 1,607  = 1,725  (data taken from [31]) | 90k  + 68k  = 158k  (data taken from [31]) | Discharge summaries &  clinical notes  (nephrology) | **Annotation Types**  **(Annotated items)**  17 Named entity types  [*Medical condition* (11,6k), *Measurement* (5,9k), *Body part* (5,4k), *Treatment* (5,3k), *Diagnostic Procedure* (4,2k), *State of Health* (4,1k), *Process* (3,9k), *Medica-tion* (3,5k), *Time* (3,4k), *Location* (2,1k), *Biochemistry* (1,8k), *Bioparameter* (1,6k), *Dosing* (1,3k), *Person* (1,3k), *Medical specification* (1,2k), *Medical device* (1,2k), *Body Fluid* (0,6k)]  (Σ: 58,6k)  10 Relation types  [*hasMeasure* (4,0k), *hasState* (3,5k), *isLocated* (2,9k), *hasTime* (2,4k), *Involves* (1,9k), *Shows* (1,5k), *has-Dosing* (0,9k), *isSpecified* (0,8k), *Examines* (0,7k), *Severity* (0,1k)]  (Σ: 18,8k)  **Entity Normalization:** N  **Annotation Guideline:** N (scheme only)  **IAA Measurement:**  N | ⚫  ◆  Information extraction model^[[5]](#footnote-5)^ |
| **Grundel-21**  [51] – 2021 | 40,485 | noi | Discharge summaries  (ophthalmology) | **Annotation Types**  **(Annotated items)**  Extraction of *Visus* (*visual acuity*; 47,6k), *Tensio* (*intraocular pressure*; 40,4k) and *Diagnoses for macular diseases* (3,2k)  **Entity Normalization:** Y (SNOMED-CT)  **Annotation Guideline:** n/a (extracted from EHR as gold standard)  **IAA Measurement:**  n/a | ⚫ |
| **Bronco**  [11] – 2021 | 200  set of  11,4k shuffled sentences | 90k | Discharge summaries (oncology: hepatocellular carcinoma or melanoma) from two national hospitals (Berlin, Tübingen) | **Annotation Types**  **(Annotated items)**  *Section Headings*  **Entity Normalization:** N  **Annotation Guideline:** Y (see Supplement)  **IAA Measurement:**  Y  3 named entity types  [*Diagnosis* (5,2k), *Treatment* (3,9k), *Medication* (2,0k)]  (Σ: 11,1k)  **Entity Normalization:** Y (ICD-10 for *Diagno-sis*, OPS for *Treatment*, ATC for *Medication*)  **Annotation Guideline:** Y (see Supplement)  **IAA Measurement:**  Y  3 types of Attributes  [*Laterality*: left, right, both-sided (1,3k), *Negation* (0,6k), *Speculation* (0,6k), *Possible Future Event* (0,6k)]  (Σ: 3,1k)  **Entity Normalization:** N  **Annotation Guideline:** Y (see Supplement)  **IAA Measurement:**  Y | **✓**  (DUA)^[[6]](#footnote-6)^ |
| **Cardio-Anno**  [46] – 2021 | 204  subset from  ~200,000 | 382k  subset from  ~218m | Discharge summaries (cardiology) | **Annotation Types**  **(Annotated items)**  12 cardiovascular concepts  [*angina pectoris* (0,2k), *dyspnea* (0,2k), *nycturia* (0,1k), *edema* (0,1k), *palpitation* (0,1k), *vertigo* (0,1k), *syncope* (0,2k), *arterial hypertension* (0,2k), *hypercholes-terolemia* (0,1k), *diabetes mellitus* (0,1k), *familial anamnesis* (0,1k), *nicotine consumption* (0,1k)]  (Σ: 1,6k)  **Entity Normalization:** Y (ICD-10)  **Annotation Guideline:** Y (see Supplement)  **IAA Measurement:**  Y | ⚫ |
| **Med-CorpInn**  **Med-CorpInn_sub_**  **Karbun**  [52,53] – 2022 | 5,003k  333k  100k | noi  61,117k    7,800k | Radiology reports | none | ⚫ |
| **Madan-22**  [54] – 2022 | 150  510  subset from  30k | noi  noi | Discharge summaries (Psychiatry: Mental Status Examination (MSE) reports) | **Annotation Types**  **(Annotated items)**  *psychiatric attributes* (3,4k), *normal* (1,7k) and *pathological assessments* (1,3k), and grounding of *pathological assessments* in the AMDP terminology (1,3k)  (Σ: 7,7k)  **Entity Normalization:** Y (ICD-10 & AMDP)  **Annotation Guideline:** Y (see Supplement)  **IAA Measurement:**  N  unlabelled | ⚫ |
| **[Ex4-CDS]**  [12] – 2022 | 720 | 13,4k*  (*estimated from averages) | Physicians’ justifications supporting their estimated likelihood of future possible negative patient outcomes after transplantation  (kidney disease endpoints: rejection, death-censored graft loss, and infection within the next 90 days) | **Annotation Types**  **(Annotated items)**  *Risk* score [0 ... 100]  (Σ: 0,4k)  4 temporal entity types  [*past*, *past-to-present*, *present*, *future*]  12 named entity types  [*Condition* (1,3k), *Diagnostic Procedure* (0,1k), *Lab Value* (0,6k), *Age of Patient/Donor* (0,1k), *Medication* (0,3k), *Process* (0,2k), *Time* (0,4k), etc.]  (Σ: 4,2k)  3 relation types  [*hasMeasure* (0,7k), *hasState* (0,4k), *hasTimeInfo* (0,3k)]  (Σ: 1,4k)  6 factuality attributes  [*Positive*, *negated* (0,3k), *speculated* (0,1k), *unlikely* (< 0,1k), *minor* (< 0,1k), and *possible future* (0,1k)]  (Σ: 0,6k)  5 progression categories  [*risk factor*, *symptom*, *increase*, *decrease*, *conclusion*]  **Entity Normalization:** N  **Annotation Guideline:** N (scheme only)  **IAA Measurement:**  Y | **✓**^[[7]](#footnote-7)^ |
| **Roller-22**  [33] – 2022  (updated version of [32]) | 61  + 1,300  = 1,361 | 57,2k  + 54,2k  = 111,4k | Discharge summaries &  clinical notes  (nephrology:  kidney transplantations) | **Annotation Types**  **(Annotated items)**  17 Named entity types  [*Medical condition* (9,0k), *Measurement* (5,4k), *Body part* (3,4k), *Treatment* (4,4k), *Diagnostic Procedure* (3,2k), *State of Health* (4,0k), *Process* (2,7k), *Medica-tion* (3,2k), *Time* (3,1k), *Location* (1.7k), *Biochemistry* (1,4k), *Bioparameter* (1,0k), *Dosing* (1,2k), *Person* (1,3k), *Medical specification* (0,9k), *Medical device* (0,4k), *Body Fluid* (0,2k)]  (Σ: 46,4k)  2 Concept Attribute types  [*DocTime*: past, past-present, future, *Factuality*: negative, speculated, unlikely, possible future]  9 Relation types  [*hasMeasure* (3,8k), *hasState* (2,9k), *isLocated* (2,2k), *hasTime* (2,3k), *Involves* (2,0k), *Shows* (1,2k), *hasDosing* (1,2k), *isSpecified* (0,6k), *Examines* (0,4k)]  (Σ: 16,6k)  **Entity Normalization:** N  **Annotation Guideline:** Y (scheme only)  **IAA Measurement:**  Y  PoS (STTS tag set), dependency parse trees [115] | ⚫  ◆  Information extraction model^[[8]](#footnote-8)^  (DUA) |
| **Trienes-22**  [55] – 2022 | 851 | 327k  (expert)  463k  (simplified)  Σ: 790k | pathology reports of sarcoma patients | Parallel corpus of expert-level and layman-directed, patient-friendly parallel versions of pathology reports | ⚫  (efforts for data sharing under way) |
| **Cardio:DE**  [45] – 2023 | 500 | 993k | clinical notes and reports (cardiology: 311 in-patient &  172 out-patient letters, and 17 letters of the cardiac emergency room) | **Annotation Types**  **(Annotated items)**  14 named entity types for section headings  [*salutation* (0.5k), *anamnesis* (1,5k), *diagnosis* (*admis-sion/discharge*; 9,8k), *medication* (*admission/dis-charge*; 7,8k), *findings* (19,3k), *lab data* (67,6k), *risk factors/allergies* (1,3k), *final recommendation* (4,5k), *summary* (3.5k), etc.]  (Σ: 116,9k)  **Entity Normalization:** Y (CDA-compliant)  **Annotation Guideline:** Y (see Supplement)  **IAA Measurement:**  Y  2 named entity types for medication & 7 relation types  [*Active Ingredient* (7,6k) or *Drug* (2,1k), *Dosage* (0,2k), *Duration*(1,5k), *Form* (0,2k), *Frequency* (6,5k), *Reason* (1,5k), *Route* (0,6k), *Strength* (6,4k)]  (Σ: 24,2k (26,6k), with 15,1k medication relations)  **Entity Normalization:** N  **Annotation Guideline:** Y (see Supplement)  **IAA Measurement:**  Y | **🖂**  **✓**  (DUA based on patient consent)^[[9]](#footnote-9)^ |
| **Llorca-23**  [56] – 2023 | 150  < 500  30  (10.2k text segments)  63 | 71k  800k  1,877k  43k | Discharge summaries (oncology) from Bronco  Discharge summaries (cardiology) from Cardio:DE  Clinical guidelines (oncology) from GGPOnc 2.0  Synthetic discharge summaries and case reports from GraSCCo 1.0 | Harmonizing approach for four German medical corpora (Bronco, Cardio:DE, GGPOnc 2.0, GraSCCo 1.0) using the BigBIo framework [94]:   - harmonizing different technical data formats (JSon, Brat/BioC, etc.), - harmonizing references to various terminologies (e.g., terms grounded in Snomed CT or different versions of ICD), - defining annotation mappings among “similar” named entities for entity alignment, and - coping with different types of entity spans   **Entity Normalization:** Y (SNOMED CT, ICD-10)  **Annotation Guideline:** n/a  **IAA Measurement:**  n/a | **🖂**  **✓**  (DUA)^[[10]](#footnote-10)^  **✓**  (public) |
| **GeMTeX**  [57] – 2023 | > 150k |  | Clinical reports covering  4 medical areas  (cardiology, pathology, pharmacy, and neurology)  from 6 different clinical sites  (e.g., discharge summaries, findings reports) | **Annotation Types**  Multiple annotation layers  **Entity Normalization:** Y (SNOMED CT, ICD-10; planned)  **Annotation Guideline:** Y (not reported)  **IAA Measurement:**  Y (not reported) | **🖂**  **✓**  (DUA  based on a broad consent model) |
| **3000PA 5.0**  [39] – 2024 | J: 1,106  A: 1,715  L: 3,823  Σ=6,644 | J: 1,8m  A: 1,7m  L: 3,8m  Σ= 7,3m | Clinical reports  from 3 different clinical sites (Jena, Aachen, and Leipzig) – (mainly discharge summaries and transfer reports) | Automatic tagging with token and sentence boundaries (silver standard)  **Annotation Types**  **(Annotated items)**  Textual macrostructure segment information – section headings such as *Family & Patient Anamnesis*, *Medication*, *Diagnosis*, etc. (Σ: 268k)  **Entity Normalization:** N (CDA-compliant)  **Annotation Guideline:** Y^[[11]](#footnote-11)^  **IAA Measurement:**  Y (not reported)  Named entities such as *Medications*, *Signs and Symptoms*, *Findings*, *Diagnoses*, and *PII* (Σ: 1,443k)  **Entity Normalization:** N  **Annotation Guideline:** Y^[[12]](#footnote-12)^  **IAA Measurement:**  Y (not reported)  Semantic relations between named entities  (Σ: 135k)  **Entity Normalization:** N  **Annotation Guideline:** N  **IAA Measurement:**  Y (not reported)  Temporal relations between named entities  (Σ: 107k)  **Entity Normalization:** N  **Annotation Guideline:** N  **IAA Measurement:**  Y (not reported)  Certainty information, including negation  (Σ: 141k)  **Entity Normalization:** N  **Annotation Guideline:** N  **IAA Measurement:**  Y (not reported)  Σ_all_: 2,093k annotated items | ⚫ |
| **Bressem-24**  [49] – 2024 | 2,000  + 2,000  + 2,000  = 6,000  subset from  3,7m  radiology reports  4,369  62  63,884  11,322  12,139  257,999  330,994  373,421  7,486  3,639  Σ 4,723,010 | 854k*  (*estimated)  520,718k  + 1,194k  + 44k  + 12.299k  + 9,324k  + 1,984k  + 259,285k  + 186,201k  + 69,639k  + 90,381k  + 2,800k  Σ 1,155,946k | Radiology reports  (chest radiographs, chest CT scans, CT/radiograph examinations of the wrist covering a wide range of bone, lung, heart, and vascular diseases  *Additional corpus resources provid-ed for training the medBERT model:*  GGPOnc 2.0  GraSCCo  DocCheck Flexikon:  Open wiki about diseases, diagnostic procedures, or treatments in all areas of medicine  Webcrawl: documents from several German medical forums  German PubMed abstracts  Springer Nature: OA articles  Thieme Publishing Group:  medical textbooks and journals for continuing medical education  Electronic health records  from the Department of Nephrol-ogy and the Center for Kidney Transplantation at Charité:  Discharge summaries & surgery reports  PhD theses from the Charité  Wikipedia: Medical entries | **Annotation Types**  **(Annotated items)**   - presence/absence of 4 *pathologies* and 4 types of *therapy devices*, - presence/absence of 23 *chest patholo-gies*, - presence/absence of 42 named entity labels   **Entity Normalization:** N  **Annotation Guideline:** N  **IAA Measurement:**  N  3 named entity types  [SNOMED-CT top-level hierarchies:  *Finding*, *Substance*, *Procedure*]  (Σ: 246,5k, short-span, Σ: 201,8k, long-span)  Named entity types (self-supplied)  (Σ: 5,8k)  **Entity Normalization:** N  **Annotation Guideline:** N  **IAA Measurement:**  N  Codes extracted from the hospital informa-tion system  **Normalization:** Y (ICD-10 for diag-noses, OPS for procedures)  **Annotation Guideline:** n/a  **IAA Measurement:**  n/a | ⚫ \|  (**✓**)  pretrained model weights for medBert & radiology bench-marks)^[[13]](#footnote-13)^  **✓**(DUA)  **✓**(public)  **✓**(public)  **✓**(public)  **✓**(public)  **✓**(licenses permitting)  **✓**(licenses permitting)  ⚫  **✓**(public)  **✓**(public) |
| **Böhrin-ger-24**  [14] – 2024 | 100  + 100  + 100  = 300 | noi | ophthalmologic physicians’ letters from three different German hospitals | **Annotation Types**  771  + 1226  + 809  = 2,806 *diagnoses*  (manually curated silver standard composed of ICD-10 codes)  **Entity Normalization:** Y (ICD-10)  **Annotation Guideline:** N  **IAA Measurement:**  N | ⚫  (**🖂**)  (**✓**)  (upon request)^[[14]](#footnote-14)^ |
| **Idrissi-Yaghir-24**  [58] – 2024  **RadQA**  [58] – 2024 | 25,023k  29,273  (question-answer pairs) | 3,060,845k  noi | different types of clinical reports, clinical notes, and doctor’s letters  question-answer pairs created from 1,223 radiology reports of brain CT scans | none  one custom question for every third report (covering ~400 reports)  **Entity Normalization:** N  **Annotation Guideline:** N  **IAA Measurement:**  N | ⚫  ⚫ |
| **DMP “Herz-Mobil”** [59] – 2024 | 35,579 | 1.245k*  (estimated from mean length) | Clinical notes | **Annotation Types**  **(Annotated items)**  9 PII types: First and last Names of Health-care professional (21,9k), Patient (16,1k), other Person (7,0k), Medical site (3,2k), Website URL (> 0,0k), Email address (> 0,0k), Physical address (0,1k), Phone number (0,5k), ZIP code (0,1k)  (Σ_all_: 49k (silver standard))  **Entity Normalization:** N  **Annotation Guideline:** N  **IAA Measurement:**  N | ⚫ |
| **Plag-witz-24** [60] – 2024 | 498 | noi | Cardiac magnetic resonance imaging (MRI) reports | **Annotation Types**  **(Annotated items)**  Attribute-value pairs for 14 *cardiac function indicators*, such as *ejection fraction* or *volumes* *for the left and right ventricle*  **Entity Normalization:** N  **Annotation Guideline:** N  **IAA Measurement:**  N | ⚫ |

**Table S2:** Real Clinical Corpora for the German Language

| **Corpus /**  **Citation – Year** | **Docu-ments** | **Tokens**  (in 1,000=1k) | **Clinical**  **Document Types**  **(Text Genres)** | **Metadata** | **Avail-ability**  ⚫ **Corpus**  ◆ **Model** |
| --- | --- | --- | --- | --- | --- |
| **Becker-16**  [61] – 2016 | 61  + 54  + 42  + 42  = 199 | noi | (Mixed) clinical reports:  discharge summaries,  ECG reports,  echo reports, and  radiology reports  (taken from the ShARe/CLEF eHealth 2013 Shared Task 1 (MIMIC II) [95] 🡪 automatic translation from English to German using Google Translate) | **Annotation Types**  **(Annotated items)**  *Disorders*  (Σ: 2,8k UMLS CUIs)  **Entity Normalization:** Y (UMLS CUIs 🡪 SNOMED-CT)  **Annotation Guideline:** N (re-use of ShARe/ CLEF eHealth 2013 Shared Task gold data)  **IAA Measurement:**  N (re-use …) | **✓**  (public) |
| **N2c2-German 1.0**  [63,64] – 2022, 2023 | 303 (train)  + 202 (test)  = 505 | 173k  (train) | discharge summaries  [taken from the n2c2 2018 Shared Task Track 2 (MIMIC III) [96] 🡪 automatic translation from English to German using a pretrained neural machine translation model from *fairseq* & alignments from *fast-align* | **Annotation Types**  **(Annotated items)**  1 *Medication* entity + 6 *Medication* relation types  [*Drug* (8,3k) – *Strength* (4,1k), *Route* (4,5k), *Frequency* (5,2k), *Duration* (3,4k), *Form* (4,2k), *Dosage* (0,4k)]  (Σ: 30,2k)  **Entity Normalization:** N  **Annotation Guideline: N** (re-use of n2c2 2018 Shared Task Track gold data)  **IAA Measurement:**  N (re-use …) | **✓**  (public)  ◆  NER model^[[15]](#footnote-15)^ |
| **N2c2-German 2.0**  [62] – 2023 | 404 | 367k | discharge summaries  [taken from the n2c2 2018 Shared Task Track 2 (MIMIC III) [96] 🡪 automatic translation from English to German using a pretrained neural machine translation model from *fairseq* & alignments from *Awesome-Align* | **Annotation Types**  **(Annotated items)**  1 *Medication* entity + 5 *Medication* relation types  [*Drug* (26,0k) – *Strength* (10,5k), *Frequency* (9,8k), *Duration* (1,0k), *Form* (10,5k), *Dosage* (6,7k)]  [Remark: *Route* (8,6k), *Reason* (6,2k), *ADE* (1,6k) were removed from the final corpus]  (Σ: 63.4k, without overlaps (longest span preserved); 64,5k, including overlaps)  (Σ: 80,9k: pre-final, full corpus)  **Entity Normalization:** N  **Annotation Guideline: N** (re-use of n2c2 2018 Shared Task Track gold data)  **IAA Measurement:**  N (re-use …) | **✓**  (public)  ◆  NER model^[[16]](#footnote-16)^ |
| **Idrissi-Yaghir-24**  [58] – 2024 | noi  6,000k  (abstracts) | 695,000k  1,700,000k | MIMIC III clinical notes &  PubMed articles  automatic translation from English to German using a pretrained neural machine translation model from *fairseq* | none  none | ◆  Trans-lation-based model^[[17]](#footnote-17)^ |

**Table S3:** Translated Real Clinical Corpora for the German Language

| **Corpus /**  **Citation – Year** | **Docu-ments** | **Tokens**  (in 1,000 = 1k) | **Clinical**  **Document Types**  **(Text Genres)** | **Metadata** | **Avail-ability**  ⚫ **Corpus**  ◆ **Model** |
| --- | --- | --- | --- | --- | --- |
| **JSynCC 1.0**  [65] – 2018 | 399  + 468  = 867 | 193k  + 119k  = 313k | Operative reports  (orthopedics, trauma & general surgery)  Case reports/descriptions  (emergency and internal medicine, general surgery, anesthetics, ophthalmology)  [taken from e-book versions of medical textbooks, *manually* generated] | **Annotation Types**  Sentence & token splits, parts of speech (PoS)  **Entity Normalization:** Y (medically adapted STTS for PoS annotation)  **Annotation Guideline:** n/a  **IAA Measurement:**  n/a | **✓**  (public code base for re-building JSynCC  for e-book license holders)^[[18]](#footnote-18)^ |
| **GraSCCo 1.0**  [66] – 2022 | 63 | 44k | Discharge summaries  [*manually* generated from real mixed-domain clinical (hospitals in Germany and Austria) and published Web resources]  Case reports  [from Open Access journals] | none | **✓**  (public)^[[19]](#footnote-19)^ |
| **Frei-23**  [67] – 2023 | (9,845  sen-tences) | 121k | (sentences *automatically* generated via few-shot prompts (12 manually created sentences) from a large language model:  *GPT NeoX* from *EleutherAI*) | **Annotation Types**  **(Annotated items)**  3 named entity types (automatically generated silver standard)  [*Medication* (9,9k), *Dose* (7,5k), *Diagnosis* (6,0k)]  (Σ: 23.4k silver items)  **Entity Normalization:** N  **Annotation Guideline:** n/a  **IAA Measurement:**  n/a | **✓**  (public)  ◆  NER model^[[20]](#footnote-20)^ |
| **JSynCC 2.0**  [39] – 2024 | 399 | 200k | Operative reports  (orthopedics, trauma & general surgery)  Case reports/descriptions  (emergency and internal medicine, general surgery, anesthetics, ophthalmology)  [taken from e-book versions of medical textbooks, *manually* generated] | **Annotation Types**  **(Annotated items)**  Named Entities  [*Findings*, *Diagnoses*, *Procedures*, *PII*]  (Σ: 343,2k)  **Entity Normalization:** N  **Annotation Guideline:** N  **IAA Measurement:**  N | **✓**  (public code base for rebuil-ding JSynC) |
| **GraSCCo 2.0**  [39] – 2024 | 63 | 44k | Discharge summaries  [*manually* generated from real mixed-domain clinical (hospitals in Germany and Austria) and published Web resources]  Case reports  [from Open Access journals] | **Annotation Types**  **(Annotated items)**  Named Entities and Semantic Relations, Temporal Relations, Certainty, Negation  (Σ: 177,8k)  **Entity Normalization:** N  **Annotation Guideline:** N  **IAA Measurement:**  N | **✓**  (public) |
| **GraSCCo 3.0_PII_**  [17] – 2024 | 63 | 44k | Discharge summaries  [*manually* generated from real mixed-domain clinical (hospitals in Germany and Austria) and published Web resources]  Case reports  [from Open Access journals] | **Annotation Types**  **(Annotated items)**  19 PII types  [*Name – Patient* (0,2k), *Doctor* (0,2k), *Title* (0,1k), etc.,  *Date* (0,7k), *ID* (0,1k)  Location – *City* (< 0,1k), *ZipCode* (0,1k), *Street* (< 0,1k), *Hospital* (< 0,1k),  Contact – *Phone* (< 0,1k), etc.]  (Σ: 1,4k)  **Entity Normalization:** N  **Annotation Guideline:** Y^19^  **IAA Measurement:**  Y | **✓**  (public)^[[21]](#footnote-21)^ |

**Table S4:** Synthetic Clinical Corpora for the German Language

| **Corpus /**  **Citation – Year** | **Docu-ments** | **Tokens**  (in 1000 = 1k) | **Medical**  **Document Types**  **(Text Genres)** | **Metadata** | **Avail-ability**  ⚫ **Corpus**  ◆ **Model** |
| --- | --- | --- | --- | --- | --- |
| **Brown-02**  [68] – 2002 | 531,690 (journal article titles) | ~ [4,000-5,000]k | Parallel corpus (English–German) of paired journal article titles retrieved from PubMed | none | **✓** |
| **Much-More**  [69] – 2002 | ~ 9,000  (abstracts for each language) | ~ 1,000k | Parallel corpus (English–German) of abstracts from 41 medical journals hosted at the Springer Web site covering various medical subdomains (e.g. neurology, radiology) | **Annotation Types**  Sentences, tokens, parts of speech (PoS), morphological segmentation, phrasal chunks  (automatically generated silver standard)  **Lexical Normalization:**  (UMLS Specialist Lexicon)  **Annotation Guideline:** n/a  **IAA Measurement:**  n/a  term mapping to MeSH codes (subset of UMLS Metathesaurus) & semantic relations from the UMLS Semantic Network  (automatically generated silver standard)  **Entity Normalization:** Y (UMLS & EuroWordNet)  **Annotation Guideline:** n/a  **IAA Measurement:**  n/a | **✓** |
| **Sprin-ger-Link**  [70] – 2003 | 5,271 | ~ 910k | Abstracts of German medical jour-nal publications, available from an online library for medicine (SpringerLink) | **Annotation Types**  Automatically derived index terms  **Entity Normalization:** Y (local dictionary linked with MeSH terms)  **Annotation Guideline:** n/a  **IAA Measurement:**  n/a | **✓** |
| **Sprin-ger**  **Med-Title**  [71] – 2004 | 9,640 | ~ 450k  [30k sentences]  ~ 5,500k  [549k sentences] | titles plus abstracts of medical journal articles from Springer, each in German (& in English);  paired titles of medical journal articles (from PubMed) | none | **✓** |
| **FraMed**  [21] – 2004 | noi  (~6,500 sentences) | 100k | Various clinical report types (discharge, pathology, histology, and surgery reports),  a medical textbook, and  Web documents taken from a consumer health care portal (netdoktor) | **Annotation Types**  Sentence & token splits, parts of speech (PoS)  **Entity Normalization:** Y (medically adapted STTS for PoS annotation)  **Annotation Guideline:** N  **IAA Measurement:**  Y | ⚫  ◆  FraMed model as part of JCoRe  [92,93] |
| **Morin-12**  [72] – 2012 | 103  118 (English)  130 (French) | 220k  265k (English)  265k (French) | Multilingual comparable corpus (English, French, German) from scientific paper websites, with hits for “*breast cancer”* *(‘cancer du sein’* in French and *‘Brustkrebs’* in German) in titles & keyword sections only | none | **✓** |
| **Mantra [Silver]**  [73] – 2014 | Σ_EFGSD_: 4,255k  719k  + 141k  + 121k  = 981k | Σ_EFGSD_: 60,424k  5,997k  + 2,100k  + 5,194k  =13,291k | Multilingual parallel corpus: English, French, German, Spanish, Dutch), including  Medline titles (PubMed)  Drug labels (EMEA)  Patent claims (EPO) | **Annotation Types**  **(Annotated items)**  Automatically generated named entities from ensembles of NER taggers (silver standard) – mapped to UMLS CUIs and semantic groups, such as *Activities & Behaviors*, *Anatomy, Chemicals & Drugs, Devices, Disorders, Geographic Areas, Living Beings, Objects, Phenomena, Physiology*  (Σ: 75,2k for German; harmonized by threshold)  (Σ_all_: > 221k, in total)  **Entity Normalization:** Y (UMLS – MeSH, SNOMED CT, MedDRA)  **Annotation Guideline:** n/a  **IAA Measurement:**  n/a | **✓** |
| **Mantra GSC**  [74] – 2015 | Σ_EFGSD_: 1,450  + 100  + 100  + 50  = 250  (Subset of [73]) | Σ_EFGSD_: 29,329  947  + 1,956  + 3,117  = 6,020  (Subset of [73]) | Multilingual parallel corpus: English, French, German, Spanish, Dutch), including  Medline titles (PubMed)  Drug labels (EMEA)  Patent claims (EPO) | **Annotation Types**  **(Annotated items)**  named entities (gold standard) – mapped to UMLS CUIs and semantic groups, such as *Anatomy, Chemicals & Drugs, Devices, Disor-ders, Geographic Areas, Living Be-ings, Objects, Phenomena, Physiol-ogy, Procedures*  (Σ: 1,082 for German)  (Σ_all_: 5,530, in total)  **Entity Normalization:** Y (UMLS – MeSH, SNOMED CT, MedDRA)  **Annotation Guideline:** Y (see Supplement; broken link)  **IAA Measurement:**  Y | **✓** |
| **HimL 1.0**  [75] – 2017 | 781k  + 33k  + 1,848k  = 2,662k | > 60,000k  (estimated) | Multilingual parallel corpus (English, German), including  EMEA (European Medicines Agency) documents  MuchMore segments  Marec patent documents | none | **✓**  (upon request) |
| **EFSG-UVigo-MED**  [76] – 2018  **ML–UVigo-MED** | 2,130  Σ_all_: 19,210  3,147  Σ_all_: 23,647 | ~ 500k | Multi-lingual corpus:  Medline/PubMed abstracts (English, French, Spanish, German) about 26 types of *Diseases*  **Wikipedia** articles (German, English, French, Spanish, Italian, Galician, Romanian, Slovene, and Icelandic) about Human Medicine (including 22 subcategories, such as Cardiology, Endocrinology, Human Genetics, Geriatrics, Nephrology, Neurology, Oncology, Surgery and Urology, Rheumatology) | Index terms extracted from Medline  **Entity Normalization:** Y (MeSH)  **Annotation Guideline:** n/a  **IAA Measurement:**  n/a | **✓**  **✓** |
| **Villena-20**  [77] – 2020 | 59,539  Σ_all_: 93,969 | 20,438k  Σ_all_: 83.869k | (Web-scraped) Multilingual corpus (German, English, Spanish), with a 63% share of German-language medical full-text articles/abstracts | none | **✓**  Zenodo^[[22]](#footnote-22)^ |
| **GGPOnc 1.0**  [79] – 2020 | 25  (4.2k annotated text segments)  Subset of 8.4k text segments | 664k  Subset of  1,340k | (all) Clinical Practice Guidelines of the German Cancer Society (oncology) | **Annotation Types**  **(Annotated items)**  7 named entity types  [UMLS Semantic Groups: *Anatomical Structure, Chemicals & Drugs, Devices, Disorders, Living Being, Physiology, Procedures*]  1 attribute type: *TNM*  [silver standard, manually validated]  (Σ: 73,8k) [96]  **Entity Normalization:** Y (UMLS)  **Annotation Guideline:** N  **IAA Measurement:**  Y  plus evidence-based recommendation meta-data, such as *Type of Recommenda-tion*, *Recommendation Grade, Strength of Consensus, Expert Opinion, Level of Evidence, Literature References*, etc. | **✓**  (DUA)^[[23]](#footnote-23)^ |
| **GGPOnc 2.0**  [78] – 2022 | 30  (5k annotated text segments)  (Subset of 10.2k text segments)  (Superset of [79]) | 830k *  (estimated)  Subset of 1,877k | (all) Clinical Practice Guidelines of the German Cancer Society (oncology) | **Annotation Types**  **(Annotated items)**  3 named entity types  [SNOMED-CT top-level hierarchies:  *Finding* (132,8k ss\| 105,0k ls; Diagnosis or Pathology, Other Finding),  *Substance* (24,9k ss \| 18,2k ls; Clinical Drug, Nutrient/ Body Substance, External Substance),  *Procedure* (88,9k ss \| 77,7k ls; Therapeutic, Diagnostic)]  (Σ: 246,5k, short-span (ss),  Σ: 201,8k, long-span (ls))  **Entity Normalization:** Y (SNOMED-CT)  **Annotation Guideline:** Y^[[24]](#footnote-24)^  **IAA Measurement:**  Y | **✓**  (DUA)^[[25]](#footnote-25)^ |
| **BTC**  [33] – 2022 | noi  (~7,7GB) | noi | Web documents taken from a consumer health care portal &  Medical newspapers &  (German) PubMed abstracts &  Clinical case studies &  Medical textbooks | none | **✓** |
| **ChaDL**  [80] – 2022 | 50 | 32k  7,069k  + 38,374k  + 20,637k  = 66,080k | Discharge summaries (neurology)  [because of the small number of tokens & documents the clinical portion of this corpus is excluded from deeper consideration]  Drug labels  Bio-medical abstracts (LIVIVO)  Medical Wikipedia articles | **Annotation Types**  Section Headings (8 categories)  [*Header and Footer, Personal Data, Diagnoses, Anamneses, Medication, Procedures & Mea-sures, Findings, Epicrisis*]  4 named entity types  *[Medication – Dosage, Intake (medication order), Disorder, (therapeutic) Procedures, Diagnostic Measures]*  **Entity Normalization:** N  **Annotation Guideline:** Y (see Supplement)  **IAA Measurement:**  Y  none  none  none | **✓**  (access is granted to institutions adhering to trusted data privacy policies and protocols)  **✓**  **✓**  **✓** |
| **Bressem-24**  [49] – 2024 | 2,000  + 2,000  + 2,000  = 6,000  subset from  3,7m  radiology reports  4,369  62  63,884  11,322  12,139  257,999  330,994  373,421  7,486  3,639  Σ 4,723,010 | 854k*  (*estimated)  520,718k  + 1,194k  + 44k  + 12.299k  + 9,324k  + 1,984k  + 259,285k  + 186,201k  + 69,639k  + 90,381k  + 2,800k  Σ 1,155,946k | Radiology reports  (chest radiographs, chest CT scans, CT/radiograph examinations of the wrist covering a wide range of bone, lung, heart, and vascular diseases  *Additional corpus resources for training the medBERT model:*  GGPOnc 2.0  GraSCCo  DocCheck Flexikon:  Open wiki about diseases, diagnostic procedures, or treatments in all areas of medicine  Webcrawl: documents from several German medical forums  German PubMed abstracts  Springer Nature: OA articles  Thieme Publishing Group:  medical textbooks and journals for continuing medical education  Electronic health records  from the Department of Nephrology and the Center for Kidney Transplantation at Charité:  Discharge summaries & surgery reports  PhD theses from the Charité  Wikipedia: Medical entries | **Annotation Types**  **(Annotated items)**   - presence/absence of 4 *pathologies* and 4 types of *therapy devices* & - presence/absence of 23 *chest pathologies* & - presence/absence of 42 named entity labels   **Entity Normalization:** N  **Annotation Guideline:** N  **IAA Measurement:**  N  3 named entity types  [SNOMED-CT top-level hierarchies:  *Finding*, *Substance*, *Procedure*]  (Σ: 246,5k, short-span, Σ: 201,8k, long-span)  Named entity types (self-supplied)  (Σ: 5,8k)  **Entity Normalization:** N  **Annotation Guideline:** N  **IAA Measurement:**  N  none  none  none  Codes extracted from the hospital information system  **Normalization:** Y (ICD-10 for diagnoses, OPS for procedures)  **Annotation Guideline:** n/a  **IAA Measurement:**  n/a  none | ⚫ \|  (**✓**)  pretrained model weights for medBer)  **✓**(DUA)  **✓**(public)  **✓**(public)  **✓**(public)  **✓**(public)  **✓**(licenses permitting)  **✓**(licenses permitting)  ⚫  **✓**(public)  **✓**(public) |
| **Idrissi-Yaghir-24**  [58] – 2024 | ~ 6,000k  6,000k  (abstracts) | 695,000k  1,700,000k | MIMIC III clinical notes &  PubMed articles  automatic translation from English to German using a pretrained neural machine translation model from *fairseq* | none  none | ◆  Translation-based model^[[26]](#footnote-26)^ |

**Table S5:** Close Domain Proxies: Pseudo-Clinical Corpora for the German Language

| **Corpus /**  **Citation – Year** | **Docu-ments** | **Tokens**  (in 1000 = 1k) | **Medical**  **Document Types**  **(Text Genres)** | **Metadata** | **Avail-ability**  ⚫ **Corpus**  ◆ **Model** |
| --- | --- | --- | --- | --- | --- |
| **FraMed**  [21] – 2004 | noi  (~6,500 sen-tences) | 100k | Various clinical report types (discharge, pathology, histology, and surgery reports),  a medical textbook, and  Web documents taken from a consumer health care portal (netdoktor) | **Annotation Types**  Sentence & token splits, parts of speech (PoS)  **Entity Normalization:** Y (medically adapted STTS for PoS annotation)  **Annotation Guideline:** N  **IAA Measurement:**  Y | ⚫  ◆  FraMed model as part of JCoRe  [92,93] |
| **Lohr-16**  [29] – 2016 | 450  subset from  22,4k  5,8m | 266k  125,9m | Operative reports (digestive tract)  (Fragments of) newspaper articles with medical content extracted from DWDS (*Digitales Wörterbuch der Deutschen Sprache*) | **Annotation Types**  *Diagnoses*, *Procedures*  **Entity Normalization:** Y (ICD for diagnoses, OPS for executed procedures)  **Annotation Guideline:** n/a (extracted from EPR as gold standard)  **IAA Measurement:**  n/a  Mentions of 400 medical terms, such as *“patient”, “surgery”, “ambulance”*, etc.  **Entity Normalization:** N  **Annotation Guideline:** n/a  **IAA Measurement:**  n/a | **✓** |
| **EFSG-UVigo-MED**  **ML–UVigo-MED**  [76] – 2018 | 2,130  Σ_all_: 19,210  3,147  Σ_all_: 23,647 | ~ 500k | Multi-lingual corpus:  Medline/PubMed abstracts (German, English, French, Spanish) about 26 types of *Diseases*  **Wikipedia** articles (German, English, French, Spanish, Italian, Galician, Romanian, Slovene, and Icelandic) about Human Medicine (incl. 22 subcategories, such as Cardiology, Endocrinology, Human Genetics, Geriatrics, Neurology, Nephrology, Oncology, Rheuma-tology, Surgery, Urology) | Index terms extracted from Medline  **Entity Normalization:** Y (MeSH)  **Annotation Guideline:** n/a  **IAA Measurement:**  n/a  **Wikipedia** categories related to Human Medicine | **✓**  **✓** |
| **Wiki-Section** [81] – 2019 | 2,3k  (Diseases, German portion only)  subset from 38k | ~2,000k*  (*estimate)  (45.7 sentences/  article) | Wikipedia articles (German, En-glish) about *Diseases* (and *Cities*) | **Annotation Types**  **(Annotated items)**  25 topic classes  [*Diagnosis, Treatment, Symptoms, Mecha-nism, Medication, Classification,* etc.] for 6,1k headings  **Entity Normalization:** Y (Wikidata Categories (for topic classes) & BabelNet synsets (for headings))  **Annotation Guideline:** N  **IAA Measurement:**  Y  Σ_all_: 242k labeled sections and normalized topic labels for up to 30 topics | **✓**^[[27]](#footnote-27)^ |
| **TLC-Med1** [82] – 2020 | 2k  (kidney diseases)  2k  (stomach and intestines)  (Σ: 4k) | 204k  (kidney diseases)  235k  (stomach and intestines)  (Σ: 439k) | Threads from the German-language patient forum Med1 | **Annotation Types**  **(Annotated items)**  Paraphrase equivalence links be-tween medical expert (Σ: 1,7k) and medical layman expressions (Σ: 4,7k) with focus on *Symptoms, Diseases, Treatments & Examinations*  **Entity Normalization:** (UMLS & Wiktionary)  **Annotation Guideline:** N  **IAA Measurement:**  N | **✓** |
| **RSS**  [83] – 2020 | noi | 13,649k | RSS feeds about the corona-virus pandemic from 13 German news-papers and 3 non-print outlets:  print: Focus Online, Frankfurter Allgemeine Zeitung, Frankfurter Rundschau, Süddeut-sche Zeitung, Neue Zürcher Zeitung, Spiegel-Online, Standard, tageszeitung (TAZ), Die Welt, and Die Zeit;  non-print: web.de, t-online.de, & heise.de | cOWIDplus Analysis generates lexicographic metadata from RSS:   - daily and weekly frequency lists of token unigrams (POS-tagged and lemmatized) and bigrams, - daily values for the central corpus measures (redundancy, mean segmental type-token ra-tio (MSTTR), & top 100 accumu-lated token frequency share | (**✓**)  (metadata only) |
| **Beck-21**  [84] – 2021 | 3k  subset from 238k | (~555k*)  (*estimate) | Tweets  (selected by search terms, such as *Corona, Pandemic, Covid 19, Social distance*, etc.) | **Annotation Types**  **(Annotated items)**  4-category label system indicating the tweet’s stance towards govern-mental measures taken against the pandemic  [*Refute (negative; 0,3k), Support (positive; 0,5k), Comment (neutral; 1,1k), Unrelated (no measures mentioned; 1,0k)*]  (Σ: 3.0k)  **Entity Normalization:** N  **Annotation Guideline:** Y (see Appendix)  **IAA Measurement:**  Y | **✓** |
| **Fang-Covid** [85] – 2021 | 28,1k  + 13,2k  = 41,3k  (complete news arti-cle / tweet) | 22,000k*  + 10,600k*  = 32,600k*  (*estimate) | Real news articles and tweets  Fake news articles and tweets  (selected by the query terms: *Corona, Covid, Infektion, Lockdown, Impfen, Impfung, Impf-stoff*) | Automatically generated meta information relating to the articles’ spreading on social media (e.g., likes, quotes, re-tweets, replies) and user characteristics (e.g., number of followers & friends) | **✓**^[[28]](#footnote-28)^ |
| **Lifeline 1.0**  [86] – 2022 | 101  (complete forum post)  subset from 4,169 | 11,k  463k*  (*estimate) | Threads about Adverse Drug Reac-tions (ADRs) from the German-language patient forum Lifeline | **Annotation Types**  **(Annotated items)**  (Binary) categorization of documents into those reporting ADRs (101 posts) and non-ADR ones (4068 posts)  (Σ: 4.2k)  **Entity Normalization:** N  **Annotation Guideline:** Y  **IAA Measurement:**  N | **✓**  (DUA)^[[29]](#footnote-29)^ |
| **BTC**  [33] – 2022 | noi  (~7,7GB) | noi | Web documents taken from a consumer health care portal &  Medical newspapers &  (German) PubMed abstracts &  Clinical case studies &  Medical textbooks | none | **✓** |
| **ChaDL**  [80] – 2022 | 50 | 32k  7,069k  + 38,374k  + 20,637k  = 66,080k | Discharge summaries (neurology)  [because of the small number of tokens & documents the clinical portion of this cor-pus is excluded from deeper consideration]  Drug labels  Bio-medical abstracts (LIVIVO)  Medical Wikipedia articles | **Annotation Types**  Section Headings (8 categories)  [*Header and Footer, Personal Data, Diagnoses, Anamneses, Medication, Procedures & Mea-sures, Findings, Epicrisis*]  4 named entity types  *[Medication – Dosage, Intake (medication order), Disorder, (therapeutic) Procedures, Diagnostic Measures]*  **Entity Normalization:** N  **Annotation Guideline:** Y (see Supplement)  **IAA Measurement:**  Y  none  none  none | **✓**  (access is granted to institutions adhering to trusted data privacy policies and protocols)  **✓**  **✓**  **✓** |
| **Bressem-24**  [49] – 2024 | 2,000  + 2,000  + 2,000  = 6,000  subset from  3,7m  radiology reports  4,369  62  63,884  11,322  12,139  257,999  330,994  373,421  7,486  3,639  Σ 4,723,010 | 854k*  (*estimated)  520,718k  + 1,194k  + 44k  + 12.299k  + 9,324k  + 1,984k  + 259,285k  + 186,201k  + 69,639k  + 90,381k  + 2,800k  Σ 1,155,946k | Radiology reports  (chest radiographs, chest CT scans, CT/radiograph examinations of the wrist covering a wide range of bone, lung, heart, and vascular diseases  *Additional corpus resources for training the medBERT model:*  GGPOnc 2.0  GraSCCo  DocCheck Flexikon:  Open wiki about diseases, diagnostic proce-dures, or treatments in all areas of medicine  Webcrawl: documents from several German medical forums  German PubMed abstracts  Springer Nature: OA articles  Thieme Publishing Group:  medical textbooks and journals for continuing medical education  Electronic health records  from the Department of Nephrology and the Center for Kidney Transplantation at Charité:  Discharge summaries & surgery reports  PhD theses from the Charité  Wikipedia: medical entries | **Annotation Types**  **(Annotated items)**   - presence/absence of 4 *pathologies* and 4 types of *therapy devices* & - presence/absence of 23 *chest pathologies* & - presence/absence of 42 named entity labels   **Entity Normalization:** N  **Annotation Guideline:** N  **IAA Measurement:**  N  3 named entity types  [SNOMED-CT top-level hierarchies:  *Finding*, *Substance*, *Procedure*]  (Σ: 246,5k, short-span, Σ: 201,8k, long-span)  Named entity types (self-supplied)  (Σ: 5,8k)  **Entity Normalization:** N  **Annotation Guideline:** N  **IAA Measurement:**  N  none  none  Codes extracted from the hospital information system  **Normalization:** Y (ICD-10 for diagnoses, OPS for procedures)  **Annotation Guideline:** n/a  **IAA Measurement:**  n/a  none  none | ⚫ \|  (**✓**)  pretrained model weights for medBer)  **✓**(DUA)  **✓**(public)  **✓**(public)  **✓**(public)  **✓**(public)  **✓**(licenses permitting)  **✓**(licenses permitting)  ⚫  **✓**(public)  **✓**(public) |
| **Lifeline 2.0**  [87]^[[30]](#footnote-30)^ – 2024 | 118  (complete forum post)  subset from  ~10k | 29,0k | Threads about Adverse Drug Reac-tions (ADRs) from the German-language patient forum Lifeline (comparable data also available for French & Japanese) | **Annotation Types**  **(Annotated items)**  12 entity types, 4 attribute types, and 13 relation types related to ADRs:  Entities and associated attributes, e.g.,   - *Drug* (0,6k), with attributes *increase, de-crease, stopped, started, unique_dose,* - *Time*, with attributes *frequency, duration, date, point in time,* - *Disorder* (1,2k), *Route, Anatomy, (Body) Function, Test,* etc.   (Σ: 3,5k entities and 1,1k attributes)  (Σ_ent+att_: 4,6k)  **Entity Normalization:** N  **Annotation Guideline:** Y^[[31]](#footnote-31)^  **IAA Measurement:**  Y  Relations and associated entities, e.g.:   - *Caused*: drug OR disorder, dis-order OR (body) function - *Treatment_for*: drug, disorder OR (body) function - *Has_dosage*: drug, measure - *Has_result*: test, measure OR disorder OR (body) function - *Examined_with*: disorder OR Anatomy OR (body) function, test - *Interacted_with*: drug, drug - *Has_route*: drug,route   (Σ: 2,2k)  **Entity Normalization:** N  **Annotation Guideline:** Y^27^  **IAA Measurement:**  Y  (Σ_all_:6,8k)  (Binary) categorization of documents into those reporting ADRs (originally, 324 posts; 118 posts after additional (length) filtering) and non-ADR ones (9,7k posts)  **Entity Normalization:** N  **Annotation Guideline:** N  **IAA Measurement:**  N | **✓**  (DUA) |
| **Heinrich-24**  [88] – 2024 | 1099  (posts)  Subset from  > 13 million posts collected from over 200 different Telegram  channels | ~198k  Subset from  ~ 400 million tokens | Posts from Telegram on conspiracy narratives surrounding the COVID-19 pandemic | **Annotation Types**  **(Annotated items)**  14 labels for conspiracy-related or conspiracy-adjacent content,  [e.g., pseudo-pandemic, criticism of counter-measures, alternative treatments, vaccine hazards, COVID-19 conspiracies, other con-spiracies, QAnon, group-focused enmity, state as an enemy, indoctrination, esoteri-cism & pseudo-science, etc.]  (Σ: ~ 0,8k)  **Entity Normalization:** N  **Annotation Guideline:** Y (not reported)  **IAA Measurement:**  Y | **✓**  (DUA)^[[32]](#footnote-32)^ |
| **HealthFC** [89] – 2024 | 750 (health-related claims & evidence informa-tion) | ~ 675k | Bilingual corpus (English – German) for medical fact checking selected from the Web portal *Medizin Transparent* | **Annotation Types**  **(Annotated items)**  *Claim – evidence – verdict* text triples:  (Public health) *claims* automatically selected from the Web portal  (related, e.g., to eating habits, dietary topics, the immune system, the respiratory, mus-culo-skeletal, or cardiovascular systems, alternative medicine, etc.),  *evidence* sentences for each claim  (manually extracted from clinical trials or systematic reviews that were manually phrased in layman language and manually annotated, incl. medical explanations),  *verdicts* manually assembled from medical experts  (i.e., *supported* (202), *refuted* (125), *not enough information* (423))  (Σ: 750 triples)  **Entity Normalization:** N  **Annotation Guideline:** N  **IAA Measurement:**  Y | **✓**  (Git-hub)^[[33]](#footnote-33)^ |
| **Pedrini-24**  [90] – 2024 | 60  (CT summaries re-phrased in layperson language) | 145k | Parallel corpus (English, German, Italian, so altogether 180 CT sum-maries) of layperson summaries of clinical trials (CT) | none | **✓** |
| **Frei-24**  [91] – 2024 | 84,478  (text fragments) | 2,023k | Wikipedia text fragments, labelled with an *Anatomical Therapeutic Chemical* (ATC) code | **Annotation Types**  **(Annotated items)**  ATC code tags (automatically ex-tracted from WikiData)  (Σ: 105,2k codes)  **Entity Normalization:** Y (WikiData QID numbers & ATC)  **Annotation Guideline:** n/a  **IAA Measurement:**  n/a | **✓**  (Git-hub)^[[34]](#footnote-34)^ |

**Table S6:** Distant-Domain Proxies: Medical Non-Clinical Corpora for the German Language

1. The distinctions from above deserve some further clarifications. Corpora were classified as “inaccessible” if either accessibility was explicitly denied in the publication, or public distributability was not all mentioned and no de-identification efforts were reported. “Public availability” has two decision branches. The first one either relies on a *formal* procedure, mostly based on contractual DUAs, or on *informal* (private) commitments which leave open (but at the same time do not explicitly preclude) whether access will be granted. Admittedly but intentionally, this is a soft constraint for distribution permissions. The second branch, “public availability without restrictions” simply holds if the corpus comes with a valid physical address for download from a digital host (e.g., institutional directories, open resource distribution sites such as GitHub or Zenodo, etc.). [↑](#footnote-ref-1)
2. <https://julielab.de/Resources/JCoRe.html> [↑](#footnote-ref-2)
3. The corpus has been announced to be publicly available in the supplementary online files of the publication. However, upon inspection of the supplement, the corpus was not listed. Further email communication with the authors revealed that this statement was way too optimistic. In conclusion, the corpus cannot be distributed. [↑](#footnote-ref-3)
4. The **Rad-Bert** model (trained on 3,8m on-site radiology reports and a 30k radiology-specific dictionary) is distributed via GitHub: <https://github.com/rAIdiance/bert-for-radiology> [↑](#footnote-ref-4)
5. <http://biomedical.dfki.de> (this link does not direct to the language model and seems deprecated) [↑](#footnote-ref-5)
6. <https://www2.informatik.hu-berlin.de/~leser/bronco/index.html> [↑](#footnote-ref-6)
7. <https://github.com/DFKI-NLP/Ex4CDS> [↑](#footnote-ref-7)
8. <https://github.com/DFKI-NLP/mEx-Docker-Deployment> [↑](#footnote-ref-8)
9. <https://heidata.uni-heidelberg.de/> [↑](#footnote-ref-9)
10. <https://huggingface.co/datasets/bigbio/> [↑](#footnote-ref-10)
11. <https://doi.org/10.5281/zenodo.7707756> [↑](#footnote-ref-11)
12. Medications: <https://doi.org/10.5281/zenodo.7707947>; Signs and Symptoms, Findings, and Diagnoses:

    <https://doi.org/10.5281/zenodo.7707917>; PII: <https://doi.org/10.5281/zenodo.7707882> [↑](#footnote-ref-12)
13. <https://github.com/DATEXIS/medBERT.de> [↑](#footnote-ref-13)
14. Send requests to: [daniel.boehringer@uniklinik-freiburg.de](mailto:daniel.boehringer@uniklinik-freiburg.de) [↑](#footnote-ref-14)
15. <https://github.com/frankkramer-lab/GERNERMED> [↑](#footnote-ref-15)
16. <https://github.com/frankkramer-lab/GERNERMEDpp> [↑](#footnote-ref-16)
17. <https://huggingface.co/ikim-uk-essen> [↑](#footnote-ref-17)
18. Software infrastructure is available at <https://github.com/JULIELab/jsyncc> [↑](#footnote-ref-18)
19. <https://doi.org/10.5281/zenodo.6539131> [↑](#footnote-ref-19)
20. <https://github.com/frankkramer-lab/GPTNERMED> [↑](#footnote-ref-20)
21. <https://doi.org/10.5281/zenodo.11502329> [↑](#footnote-ref-21)
22. <https://zenodo.org/record/3463379.XY4RsUEzaV4> [↑](#footnote-ref-22)
23. <https://www.leitlinienprogramm-onkologie.de/projekte/ggponc-english/> [↑](#footnote-ref-23)
24. <https://github.com/hpi-dhc/ggponc_annotation> [↑](#footnote-ref-24)
25. <https://www.leitlinienprogrammonkologie.de/projekte/ggponc-english/> [↑](#footnote-ref-25)
26. <https://huggingface.co/ikim-uk-essen> [↑](#footnote-ref-26)
27. <https://github.com/sebastianarnold/WikiSection> [↑](#footnote-ref-27)
28. <https://github.com/justusmattern/fang-covid> [↑](#footnote-ref-28)
29. <https://github.com/DFKI-NLP/cross-ling-adr> [↑](#footnote-ref-29)
30. **Lifeline 2.0** contains a different set of documents than **Lifeline 1.0** (thus, they are counted as two distinct corpora) although the theme covered remains the same. [↑](#footnote-ref-30)
31. <https://github.com/DFKI-NLP/keepha_annotation_guidelines/blob/main/KEEPHA_annotation_guidelines.pdf> [↑](#footnote-ref-31)
32. <https://corpora.linguistik.uni-erlangen.de/cqpweb/schwurpus_v2/> and <https://github.com/fau-klue/infodemic> [↑](#footnote-ref-32)
33. <https://github.com/jvladika/HealthFC/> [↑](#footnote-ref-33)
34. <https://github.com/frankkramer-lab/WikiOntoNERCorpus> [↑](#footnote-ref-34)
