# Supplementary material for: Clinical document corpora—real ones, translated and synthetic substitutes, and assorted domain proxies: a survey of diversity in corpus design, with focus on German text data
Source: JAMIA Open. 2025 May 14;8(3):ooaf024. doi: 10.1093/jamiaopen/ooaf024 (PMC12077144; doi:10.1093/jamiaopen/ooaf024)
Supplement: ooaf024_Supplementary_Data [file ooaf024_supplementary_data.zip › Table_A1_Corpus_Card_OUP_ooaf024_V2.docx]

**APPENDIX A1**

| **Corpus Attributes** | **Definition** | ***Attribute Values (in italics)* & Examples** |
| --- | --- | --- |
| **Language(s)** | Natural language(s) of the document units in the corpus | - *de*: German - *en*: English - *es*: Spanish - *fr*: French, etc.   [Codes based on ISO 639] |
| **Modality** | The mode how natural language utterances are communicated or overlaid | - *written* language (textual documents) - *spoken* language (recorded speech, non-transcribed audio signals) - *visual* signals, mostly body movements (gestures, face expressions, deictic moves, etc.) - *multi-modal* – a mixture of different modalities |
| **Media** | Types of media (data) complementing natural language utterances | - *visual* data (images, photos, drawings, dia-grams, charts, figures, movies, etc.) - *structured* data (tables, etc.) - *non-speech* auditory data (music, sounds, etc.) - *sensor* signals (measured physiological data, click stream or social networking data, etc.) - *multi-media* – a mixture of various media |
| **Data status** | Status of the data, i.e., whether they are originally taken from the domain of dis-course, or whether they are purposefully altered/modified (hence, not original) | - *original* (i.e., authentic, de-identified) data - *translated* data (automatic language-to-language translations, e.g., en2de) - *synthetic* (i.e., fictitious, invented) data |
| **Corpus Versioning** | If a family of versions of basically the same or a similar corpus emerges, the relationship of a specific corpus to its predecessors should be made explicit in set-theoretical terms | - *=* (the current corpus version shares all documents with a specified reference version) - *!=* (the current corpus version shares no document with a specified reference ver-sion) - *SuperSet/SubSet-of* (the current corpus version is a superset/subset of the doc-uments of a specified reference version) - *ProperIntersection* (the current corpus version has a non-empty intersection with the documents of a specified reference version, yet is neither a subset nor a superset) |
| **# Documents** | Absolute number of document units |  |
| **# Superset** | The (size of the) superset from which a subset (= corpus) was drawn, if any | Comment: # Superset >> # Documents |
| **# Tokens** | Absolute number of single “words” |  |
| # Types | Absolute number of distinct single “words” |  |
| # Other document units | Absolute number of sentences, paragraphs, sections/chapters, segments (in parallel/ comparable corpora), etc., if any |  |
| Average length of documents | Arithmetic *mean* (incl. standard deviation) or *median* of #tokens (or other document units, if any) per document |  |
| Sampling strategy | How were the documents sampled? | - *ad hoc* sample (arbitrary, often subjective selection of data items) - *random* sample, etc. |
| Data splits | (Recommended) data splits for training, de-velopment/validation, testing in the corpus | An example:  70:15:15 |
| **Release conditions** | Distribution status of the corpus, i.e., whether the corpus is publicly sharable or not and, if so, under which access conditions | - *Non-distributable*, classified, inaccessible for external use - *Regulated* distribution on a contractual basis (e.g., DUA, IPR licence, royalties/ fees) - *Publicly* shared (e.g., on online sites such as Zenodo or GitHub), without constraints |
| Technical format | Storage format of the corpus | - UTF-8, XML, JSON, etc. |
| **Contact Data** | For *regulated* access: The digital address of the person in charge of the corpus (the cor-pus owner or administrator)  For *public* access: the digital address of the site which hosts the corpus | - An *e-mail* address - A *URI* (URL or URN) |
| **Genre Attributes** |  |  |
| **Verbal interaction mode** | Types of verbal interaction modes prevalent in the document units of the corpus | - *monologic* data: typically, written texts, with readers as addressees, such as clinical reports or notes, encyclopedia articles, scientific papers/abstracts or newspaper articles, books, etc. - *dialogic* data: typically, written or spoken utterance exchanges between two speak-ers, such as tweets, chats, posts, question-answer sequences, conversations - *multi-party* data: typically, written or spo-ken utterance exchanges between more than two speakers, e.g., in meetings, discussion groups |
| **(Medical) Document genre(s)** | Types of (medical) documents characterized by normative writing habits, conventions relating to contents, communicative goals and formal document structure, as well as type-specific linguistic style relating to choic-es of terminology, abbreviations & acronyms, phrasal patterns, etc. | Monologic:   - *clinical reports*/notes, such as discharge summaries, pathology or radiology reports, nurse notes, case reports, etc. - *clinical guidelines* - *clinical trial* *reports* - *clinical case descriptions*   Dialogic:   - *patient-doctor* conversations   Multi-party:   - oncologic *council*   Comment: For reasons of clarity and added value,   - *clinical domains*, such as vascular and casualty surgery, internal medicine, neurology, anaesthesia, intensive care, radiology, physiotherapy, and - *anatomical regions* targeted in the documents, as with lung cancer, thorax X-rays, etc.   should be co-mentioned with medical document genres |
| # Documents/  genre | Absolute number of documents per genre |  |
| Average length of documents/genre | Arithmetic *mean* (incl. standard deviation) or *median* of #tokens per genre |  |
| **Institutional Attributes** |  |  |
| # Clinical sites/ institutions | Absolute number of clinical sites from each clinical institution (represented in the corpus) | An example:  Intensive Care Unit, Children’s Hospital, Neurosurgery Dept. @ Mayo Clinic Hospital  🡪 3 clinical sites, 1 institution |
| # Clinical institutions | Absolute number of clinical institutions (represented in the corpus) | An example:  Mayo Clinic Hospital, The Vanderbilt Clinic – Nashville, Kerrville VA Hospital  🡪 3 institutions |
| # Countries / Languages | Absolute number of countries and languages (represented in the corpus) | An example:  Mayo Clinic Hospital, USA; Klinikum rechts der Isar, Germany  🡪 2 countries / 2 languages |
| **Metadata Attributes** |  |  |
| **Annotation type(s)**  **(& attribute(s))** | Clinically relevant metadata categories: Boolean and multi-valued categories, named entities and associated attributes, relations, etc. | An example:  Symptom, finding, diagnosis  🡪 3 named entity types  Drug: frequency, dosage, mode, duration  🡪 1 named entity type, 4 attributes  Smoker status: Smoker/non-smoker  🡪 1 categorical type (Boolean)  Age: infant, adolescent, adult, elderly  🡪 1 categorical type (4-valued)  Treatment_for: drug, disorder  Has_result: test, (body) function  Interacted_with: drug, drug  Time_before: disorder, disorder  🡪 4 relation types |
| **# Annotation in-stances/anno-tation type**  **(& attributes)** | Absolute number of annotated items per annotation type (and associated attributes) |  |
| **Term normalization (Grounding)** | Annotation types/instances with mappings into common (medical) terminologies, ontolo-gies, lexicons | An example:  *ICD 10*: Disease_type_ – bacterial pneumonia_instance_  🡪 J15_ICD_10_  *SNOMED CT*: Disease_type_ – Tuberculosis (disorder)_instance_  🡪 56717001_SNOMED_CT_ |
| **# Annotators + Mediators** | Absolute number of annotators (incl. educa-tional background) & mediators/managers (incl. educational background) |  |
| **IAA / Annotation type** | Scores for inter-annotator agreement (IAA) per annotation type under different matching conditions, if any, e.g.,   - strict match - sloppy (lenient) match | Comment: using metrics such as F1, Krippendorff’s α, Cohen’s κ, etc. |
| Average annotation time | Arithmetic *mean* (incl. standard deviation) or *median* of the time required to annotate all single metadata items per annotation type (for each annotator & aggregated for the en-tire annotation team, i.e., micro & macro IAA) |  |
| Technical format | Storage format of the annotations | BRAT/BioC, JSON etc. |

**Table A1:** Corpus Card – a Template Datasheet for Corpus Descriptions, with Mandatory (bold) and Optional Attributes (non-bold)

This template primarily focuses on (clinically/medically relevant) content issues only. Additional attributes may have to be added for a more comprehensive template (see, e.g.,[116,117]).
